# In Silico Characterization and Expression Analysis of *GIGANTEA* Genes in Potato

**DOI:** 10.1007/s10528-022-10214-7

**Published:** 2022-03-11

**Authors:** Flóra Karsai-Rektenwald, Khongorzul Odgerel, Jeny Jose, Zsófia Bánfalvi

**Affiliations:** Genetic and Biotechnology Institute, Hungarian University of Agriculture and Life Sciences, Szent-Györgyi A. u. 4, Gödöllő, 2100 Hungary

**Keywords:** GIGANTEA, *Solanum tuberosum*, Promoter elements, Abiotic stress response, Transcription factors

## Abstract

*GIGANTEA* (*GI*) genes are ubiquitous in the plant kingdom and are involved in diverse processes from flowering during stress responses to tuberization; the latter occurs in potato (*Solanum tuberosum* L.). *GI* genes have a diurnal cycle of expression; however, no details on the regulation of *GI* gene expression in potato have been reported thus far. The aim of our work was the analysis of the *GI* promoter sequence and studying *GI* expression in different organs and under abiotic stress conditions in potato. Two *GI* genes homologous to *Arabidopsis GI* located on chromosomes 4 and 12 (*StGI.04* and *StGI.12*) were identified in the genome-sequenced potato *S. phureja*. The *GI* promoter regions of the commercial potato cultivar ‘Désirée’ were cloned and found to be almost identical to the *S. phureja GI* promoter sequence. More than ten TF families binding to the *GI* promoters were predicted. EVENING ELEMENT and ABSCISIC ACID RESPONSE ELEMENT LIKE elements related to circadian regulation and a binding site for POTATO HOMEOBOX 20 presumably involved in tuber initiation were detected in both *GI* promoters. However, the locations of these elements and several other *cis*-acting regulatory elements as well as the organ-specific expression and responses of the genes to abiotic stresses and abscisic acid were different. Thus, we presume that the function of *StGI.04* and *StGI.12* are at least partially different. This study lays foundation for further investigation of the roles of *GI* genes in potato.

**Supplementary Information:**

The online version contains supplementary material available at 10.1007/s10528-022-10214-7.

## Introduction

In 1962, a “supervital” mutant with a late flowering phenotype and a 2.5–3 times longer life cycle, producing 25 times as much dry material and roughly ten times as many seeds as the wild type, was identified in *Arabidopsis thaliana* and designated GIGANTEA (GI) based on its phenotype (Rédei 1962). It is ubiquitous in the plant kingdom, and the evolution of GI can be proposed to have taken place alongside the origin of land plants. GI is a circadian clock-regulated protein known to have pleiotropic functions owing to its involvement in diverse processes, such as flowering time regulation, control of circadian rhythm, hypocotyl elongation, vegetative growth, chlorophyll accumulation, light-, sucrose- and hormone-signaling, starch accumulation, transpiration, herbicide-, cold- and drought-tolerance, miRNA processing and floral scent emission (reviewed by Mishra and Panigrahi 2015; Jose and Bánfalvi 2019; Brandoli et al. 2020).

Transcription of *GI* is regulated by the circadian clock, with a peak in transcript levels 8–10 h after dawn (Park et al. 1999). The timing, height and duration of this peak are influenced by the day length. The rhythmic pattern of *GI* expression is altered in *EARLY FLOWERING 3* (*ELF3*) and *LATE ELONGATED HYPOCOTYL* (*LHY*) mutants as well as in *CIRCADIAN CLOCK ASSOCIATED 1* (*CCA1*)-overexpressing *Arabidopsis* plants (Fowler et al. 1999). The activities of the clock-associated proteins TIME FOR COFFEE (TIC) and LIGHT-REGULATED WD1 and 2 (LWD1 and LWD2) are required for the repression of *GI* transcription in the morning (Hall et al. 2003; Wu et al. 2008). Furthermore, PSEUDO RESPONSE REGULATORS (PRRs) are also involved in the regulation of *GI* expression (Nakamichi et al. 2007; Kawamura et al. 2008).

Lu et al. (2012) demonstrated that CCA1 represses *GI* expression by binding to the *GI* promoter. The CCA1 binding motif is closely related to the so-called EVENING ELEMENT (EE) detected in the promoters of those genes, which are expressed late in the day. Berns et al. (2014) identified three EEs and three ABA RESPONSE ELEMENT LIKE (ABREL) elements in the *Arabidopsis GI* promoter and showed that they contribute to the light inducibility of *GI* transcription. The night time repression of *GI* transcription is attributed to the evening complex consisting of ELF3, ELF4 and LUX ARRHYTHMO (Nusinow et al. 2011; Helfer et al. 2011). In addition to light, *GI* expression is also regulated by temperature, as a warmer temperature of 28 °C upregulates *GI* transcript levels in comparison to a cooler temperature of 12 °C (Paltiel et al. 2006).

*GI* genes have been detected and isolated from several plant species from monocots to dicots and found to show diurnal cycles of expression and conserved functions in terms of flowering time determination and circadian clock regulation (reviewed by Mishra and Panigrahi 2015). *GI* has ubiquitous expression in all organs and in all stages of plant growth, but at different levels (Fowler et al. 1999; Sawa and Kay 2011; Luo et al. 2011; Tang et al. 2017).

The *GI* gene is also present in potato (*Solanum tuberosum* L). In potato, GI is involved not only in the initiation of flowering but also in the initiation of tuberization (Kloosterman et al. 2013). The wild Andean landrace *Solanum tuberosum* Group Andigena is a strict short day (SD) plant for tuberization (Jackson 2008). It was shown that the expression of *GI* is regulated by PHYTOCHROME B (PHYB) in the leaves of Andigena potato plants (Rutitzky et al. 2009). Morris et al. (2014) investigated the molecular basis of permissive tuber initiation in Neo-Tuberosum by comparative analysis with an Andigena accession and tested *GI* expression under long day (LD) and SD conditions. A diurnal cycle of *GI* mRNA levels in both genotypes under both conditions was detected; however, the peak sizes were much higher in Andigena than in Neo-Tuberosum. Independent of day length, *GI* expression peaked at 8 h after dawn.

The aim of the current study was to investigate the transcriptional regulation of the *GI* gene in the tetraploid commercial potato cultivar ‘Désirée’ both in silico and by reverse transcription-quantitative polymerase chain reaction (RT-qPCR). We show that potato harbor two copies of *GI,* which are regulated at least partially in different ways.

## Materials and Methods

### Plant Materials and Growth Conditions

The tetraploid potato (*Solanum tuberosum* L*.*) cultivar ‘Désirée’ was used as plant material in this study. Plantlets were cultivated in vitro in RM culture medium (MS medium without vitamins; Murashige and Skoog 1962) supplemented with 2% (w/v) sucrose and 0.8% agar and adjusted to pH 5.8–6.0. Plantlets were grown in a culture room at 24 °C under a photoperiod of 16 h/8 h day/night cycle at a light intensity of 75 μmol m^−2^ s^−1^. Plantlets with apical buds were continuously subcultured in RM medium every 4 weeks or propagated from stem segments carrying a single auxillary bud. Four-week-old plantlets were transferred into sterile A200 soil (Stender GmbH, Schermbeck, Germany) and grown further under greenhouse conditions with a photoperiod of 14 h day/10 h night and a temperature regime of 20–28 °C. Optimal growth conditions were provided by watering the plants twice a week. Pesticides and fungicides were applied in the greenhouse to avoid contamination. The tubers were harvested 16 weeks after transferring the in vitro plantlets into the pots. Different organs of the potato plants, including the roots, stolons, tubers, stems, petioles, leaves, sepals, petals and stamens, were collected for *GI* expression analysis, and the leaves were sampled for stress treatment experiments.

### Stress and Abscisic Acid (ABA) Treatments

Leaves of potato plants grown in pots in a greenhouse for 6–8 weeks were subjected to different abiotic stress treatments. Three to five source leaves derived from three to five plants were used for each treatment. The first leaflets of compound leaves with petioles were cut from the plants at 6 h after sunrise. The leaves via petioles were inserted into distilled water (control) in a beaker or into 200 mM NaCl, 20% PEG 6000 and 0.1 mM ABA solutions and incubated at room temperature for 6 h in the laboratory, with the exception of ABA, which was applied for 24 h under greenhouse conditions. For the cold and heat treatments, leaves in distilled water were incubated at 4 °C and 42 °C, respectively, for 6 h. After the treatments, the leaf samples were frozen in liquid nitrogen and transferred immediately to − 70 °C.

### In Silico DNA Sequence Analysis of the *StGI* Promoters

Three thousand-bp sequences upstream of the translational start sites of the *S. tuberosum* Group Phureja *GI* genes located on chromosomes 4 (Soltu.DM.04G027760) and 12 (Soltu.DM.12G007510) were retrieved from the Potato Genomic Resource Spud DB (http://solanaceae.plantbiology.msu.edu/), while the sequence of the same upstream region of *A. thaliana GI* gene (AT1G22770.1) was retrieved from TAIR (https://www.arabidopsis.org/). The Plant Regulation Data and Analysis Platform (http://plantregmap.gao-lab.org/) was used to predict the binding sites of the transcription factors.

### Isolation of the Promoter Region of *StGI* Genes

The genomic DNA from in vitro* S. tuberosum* cv. ‘Désirée’ leaves was prepared as described by Shure et al. (1983), and PCR amplification was performed using the primers listed in Suppl. Table 1. The PCR fragments obtained with the primer pairs StGI.04 -2601 FW—StGI.04 -816 R, StGI.04 -816 FW—StGI.04+65 R, StGI.12 -2837 FW—StGI.12 -1586 R and StGI.12 -1586 FW—StGI.12+59 R were cloned into pGEM-T Easy (Promega, Madison, WI, USA). The primer sequences are presented in Table S1. Sanger sequencing of the cloned PCR fragments was performed at BIOMI (Gödöllő, Hungary) and analyzed by NCBI BLASTn (https://blast.ncbi.nlm.nih.gov/Blast.cgi) and the multiple alignment tool Clustal Omega (www.ebi.ac.uk/Tools/msa/clustalo).

### Reverse Transcription-Quantitative Polymerase Chain Reaction (RT-qPCR)

*StGI* gene expression levels were assayed using RT-qPCR. Total RNA extractions of the plant samples were performed using a method described previously (Stiekema et al. 1988). RNA concentration and quality were tested in a NanoDrop spectrophotometer. Two hundred ng RNA in a 20-µl reaction volume was reverse transcribed into first-strand cDNA using the Maxima H minus First Strand cDNA Synthesis Kit with dsDNase (Thermo Scientific Molecular Biology, Waltham, MA, USA), out of which 1 µl was added to the qPCR mix. RT-qPCR assays were performed using a Light Cycler-96 thermal cycler (Roche Diagnostics GmbH, Mannheim, Germany) and a Luminaris Color HiGreen Flourescein qPCR Master Mix (Thermo Scientific Molecular Biology, Waltham, MA, USA). Reverse transcriptase minus control was applied to assess for RNA sample contamination with DNA. The control reaction was performed during the first strand cDNA synthesis by combining all components for reverse transcription except the reverse transcriptase. Expression analysis of *StGI.04* and *StGI.12* was carried out using the primer pairs StGI.04spec and StGI.12spec*.* The data were normalized to two control genes*, ACTIN* and *EF1α* (Nicot et al. 2005) as a ratio between the Cq value of the target gene and the geometric means of Cq values of the two control genes. To test the level of *StGI.04* and *StGI.12* transcript levels in different organs, samples were collected from 3 to 5 potato plants grown in a greenhouse, while responses of 3–5 leaves of 3–5 plants (one leaf per plant) were analyzed after stress treatments. In each RT-qPCR assay one biological sample representing 3–5 plants was tested in three technical replicates. The efficiency of stress treatments was monitored by testing the *Δ1-PYRROLINE-5-CARBOXYLATE SYNTHETASE* (*P5CS*; Liu et al. 2019), *α-GLUCAN, WATER DIKINASE* (*GWD*; Orzechowski et al. 2021) and *HEAT SOCK PROTEIN 20–44* (*HSP20-44*; Zhao et al. 2018) mRNA levels. The gene IDs and primer sequences are listed in Table S1. The data were analyzed with Light Cycler-96 Software version 1.1 (Roche Diagnostics GmbH, Mannheim, Germany). Statistical significance of the measurements was determined by Student’s *t*-test.

## Results

### Identification of *GI* Genes in Potato

To identify the *GI* gene(s) in potato, a search for the *A. thaliana GI* (*AtGI*) NM_102124 homologue was carried out using the nucleotide BLAST tool available at NCBI. Two transcript variants with 71.77–72.97% identity to *AtGI* were found. One of them, represented by XM_006358978.2, was located on chromosome 4 (*StGI.04*), while the other, represented by XM_006361554.2, was located on chromosome 12 (*StGI.12*). The two variants had an identity of 83.73% at the transcript level. A BLAST search was carried out in the Potato Genomic Resource Spud DB, which is based on the sequence of the doubled monoploid potato *S. tuberosum* Group Phureja (PGSC 2011; Pham et al. 2020), to obtain the promoter sequence of the two variants as a − 3.0-kb region upstream of the translation start site. The two promoter regions were compared to each other and to the *AtGI* promoter using the default setting of NCBI BLASTn. No significant similarity was found between the two potato *GI* promoters or to that of the *AtGI*.

### Isolation and In Silico Characterization of *StGI* Promoters

To isolate the putative promoter regions of *StGI* genes from the commercial potato cv. ‘Désirée’ PCR primers were designed based on the promoter sequences of Phureja *GI* genes (Table S1) and tested with ‘Désirée’ genomic DNA. Two primer pairs were designed to each gene. Both the *StGI.04* and *StGI.12* promoter regions of ‘Désirée’ could be obtained, each in two fragments, and cloned. Inserts of four clones from each cloning were sequenced. The ‘Désirée’ *StGI.04* promoter, with a few base-pair differences, was identical to that of ‘Phureja’ (Fig. S1). The *StGI.12* promoter was also highly homologous between the two potato cultivars. However, three clones had a 14-bp insertion at approximately − 1.7 kb and four clones an 8–9-bp insertion at approximately − 0.3 kb (Fig. S2). Since not all four clones had an insertion at − 1.7 kb it was concluded that ‘Désirée’ carries at least two alleles of *StGI.12*.

The Plant Regulation Data and Analysis Platform (PlantRegMap) was used to predict the transcriptional binding sites in the potato *GI* promoters. Considering the very high level of homology between the *S. tuberosum* Group Phureja and *S. tuberosum* cv. ‘Désirée’ *StGI* promoter sequences (Figs. S1 and S2) the *S. tuberosum* Group Phureja *GI* promoter regions were selected for in silico analysis because the Phureja genomic sequence is the one generally accepted as a potato standard. The PlantRegMap predicted 73 binding sites of 45 transcription factors (TFs) in *StGI.04* and 32 binding sites of 27 TFs in the *StGI.12* promoter at a threshold *p* value ≤ 1e−5 (Tables S2 and S3). These TFs belonged to 14 families in the case of *StGI.04* and 13 families in the case of the *StGI.12* promoter. The common families were BBR-BPC, bZIP, C2H2, DOF, ERF, HB-other, MIKC-MADS, M-type-MADS, MYB-related and TCP. Binding sites of TFs belonging to families B3, GATA, MYB and Trihelix were present only in the *StGI.04* promoter. The dominate TF families were bZIP and DOF. The bHLH-, HD-ZIP- and NAC-type TF-binding sites were unique to the *StGI.12* promoter; however, the dominant family was common to the *StGI.04* promoter, namely, bZIP. The diversity of the binding sites suggests that the transcriptional regulation of the two *GI* genes is not identical.

**Fig. 1:**
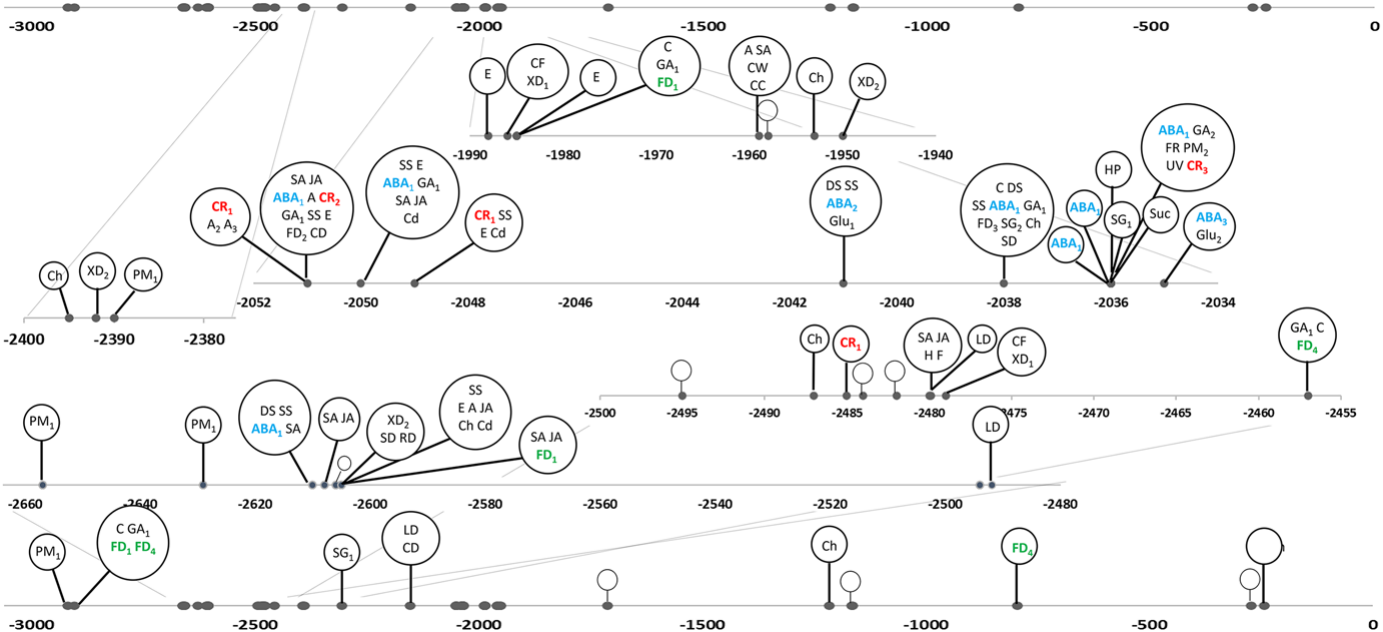
Schematic drawing of the -3-kb promoter region of the *StGI.04* gene in *S. tuberosum* Group Phureja. Both the topmost and bottom lines represent the same -3.0-kb *StGI.04* promoter region. The circles show individual transcription factors with their functions abbreviated as: *A* response to auxin; *A*_*2*_ auxin-activated signaling pathway; *A*_*3*_ regulation of auxin biosynthetic process; *ABA*_*1*_ response to abscisic acid; *ABA*_*2*_ abscisic acid-activated signaling pathway; *ABA*_*3*_ cellular response to abscisic acid stimulus, *C* response to cold, *CB* regulation of secondary cell wall biogenesis, *CC* positive regulation of cell cycle, *CD* cell differentiation, *Cd* response to cadmium ion, *CF* cell fate specification, *Ch* response to chitin, *CR*_*1*_ circadian rhythm, *CR*_*2*_ circadian regulation of gene expression, *CR*_*3*_ positive regulation of circadian rhythm, *CW* cell wall modification, *DS* response to water deprivation, *E* response to ethylene, *F* cellular response to freezing, *FD*_*1*_ positive regulation of flower development, *FD*_*2*_ photoperiodism flowering, *FD*_*3*_ pollen maturation, *FD*_*4*_ maintenance of floral meristem identity, *FD*_*5*_ petal morphogenesis, *FR* red or far-red light signaling pathway, *GA*_*1*_ response to gibberellin, *GA*_*2*_ gibberellic acid mediated signaling pathway, *Glu*_*1*_ glucose mediated signaling pathway, *Glu*_*2*_ cellular response to glucose stimulus, *H* cellular response to heat, *HP* regulation of hydrogen peroxide metabolic process, *JA* response to jasmonic acid, *LB* lignin biosynthetic process, *LD* leaf development, *OB* organ boundary specification between lateral organs and the meristem, *PM*_*1*_ photomorphogenesis, *PM*_*2*_ regulation of photomorphogenesis, *PO* plant ovule development, *RD* root development, *SA* response to salicylic acid, *SD* seed development, *SG*_*1*_ seed germination, *SG*_*2*_ negative regulation of seed germination, *SS* response to salt stress, *Suc* sucrose induced translational repression, *UV* response to UV-B, *XD*_*1*_ xylem and phloem pattern formation, xylem development, *XD*_*2*_ phloem or xylem histogenesis. Empty circles represent TFs with unknown function. The function of TFs related to the circadian rhythm, flower development and response to ABA are highlighted in red, green and blue, respectively

**Fig. 2:**
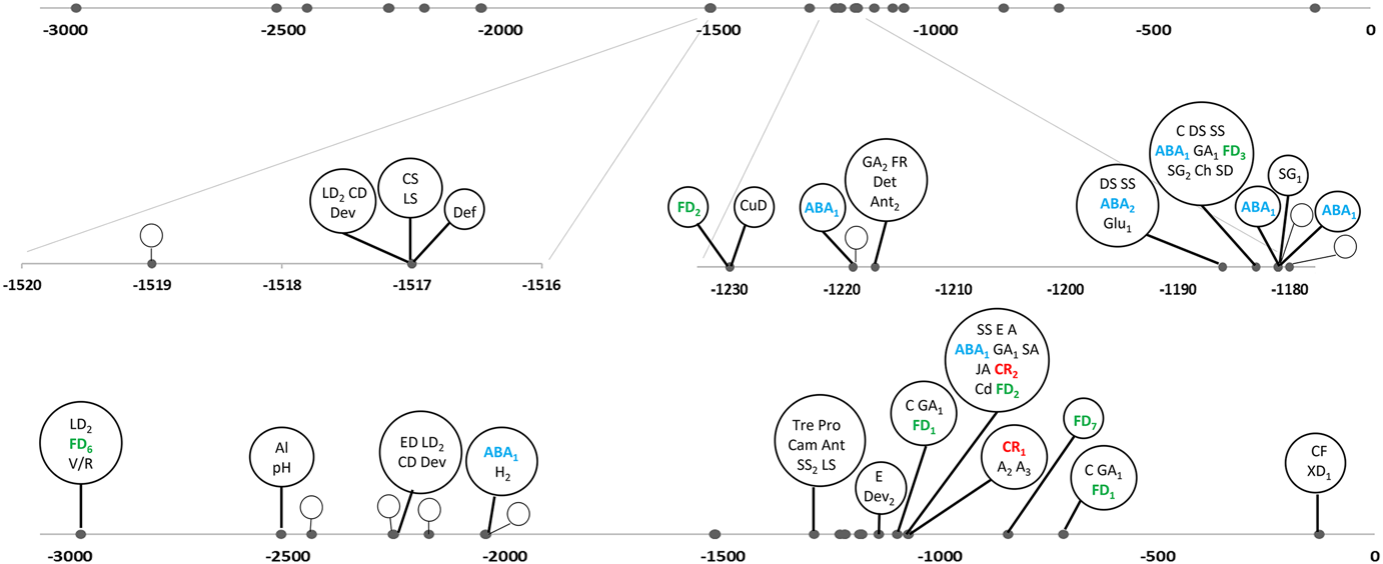
Schematic drawing of the − 3.0-kb promoter region of the *StGI.12* gene in *S. tuberosum* Group Phureja. Both the topmost and bottom lines represent the same − 3.0-kb *StGI.12* promoter region. The circles show individual transcription factors with their functions abbreviated as: *A* response to auxin, *A*_*2*_ auxin-activated signaling pathway, *A*_*3*_ regulation of auxin biosynthetic process, *ABA*_*1*_ response to abscisic acid, *ABA*_*2*_ abscisic acid-activated signaling pathway, *Al* response to aluminium ion, *Ant* anthocyanin-containing compound biosynthetic process, *Ant*_*2*_ positive regulation of anthocyanin metabolic process, *C* response to cold, *Cam* camalexin biosynthetic process, *CD* cell differentiation, *Cd* response to cadmium ion, *CF* cell fate specification, *Ch* response to chitin, *CR*_*1*_ circadian rhythm, *CR*_*2*_ circadian regulation of gene expression, *CuD* cuticle development, *CS* regulation of cell size, *Def* regulation of defense response, *Det* de-etiolation, *Dev* positive regulation of development, heterochronic, *Dev*_*2*_ regulation of developmental process, *DS* response to water deprivation, *E* response to ethylene, *ED* embryo development ending in seed dormancy, *FD*_*1*_ positive regulation of flower development, *FD*_*2*_ photoperiodism, flowering, *FD*_*3*_ pollen maturation, *FD*_*6*_ floral meristem determinacy, *FD*_*7*_ specification of floral organ identity; *FR* red or far-red light signaling pathway, *GA*_*1*_ response to gibberellin, *GA*_*2*_ gibberellic acid mediated signaling pathway, *Glu*_*1*_ glucose mediated signaling pathway, *H*_*2*_ heat acclimation, *JA* response to jasmonic acid, *LD*_*2*_ leaf morphogenesis, *LS* negative regulation of leaf senescence, *pH* response to acidic pH, *Pro* proline biosynthetic process, *SA* response to salicylic acid, *SD* seed development, *SG*_*1*_ seed germination, *SG*_*2*_ negative regulation of seed germination, *SS* response to salt stress, *SS*_*2*_ hyperosmotic salinity response, *Tre* trehalose biosynthetic process, *XD*_*1*_ xylem and phloem pattern formation, xylem development, *V/R* regulation of timing of transition from vegetative to reproductive phase. Empty circles represent TFs with unknown function. The function of the TFs related to circadian rhythm, flower development and response to ABA are highlighted in red, green and blue, respectively

The locations of the TF binding sites and the biological processes in which these TFs are involved are presented in Figs. 1 and 2 for the *StGI.04* and *StGI.12* -3.0-kb promoter regions, respectively. In both promoters, binding sites of TFs responding to circadian rhythm, salt stress, abscisic acid (ABA), ethylene, auxin, jasmonic acid, chitin and cadmium ions were identified. These sites were located at approximately − 1.4 kb in the *StGI.12* promoter but were mainly present at approximately − 2.0 kb in the *StGI.04* promoter. Binding sites for TFs involved in flower development were found in both promoters. Despite the functional similarities of the 45 and 27 TFs recognizing the *StGI.04* and *StGI.12* promoters, respectively, only 14 occurred in both. This finding supported the conclusion that the regulation of the two *GI* genes is not identical in potato.

### Detection of Similarities Between *Arabidopsis*- and Potato *GI* Promoters

**Fig. 3:**
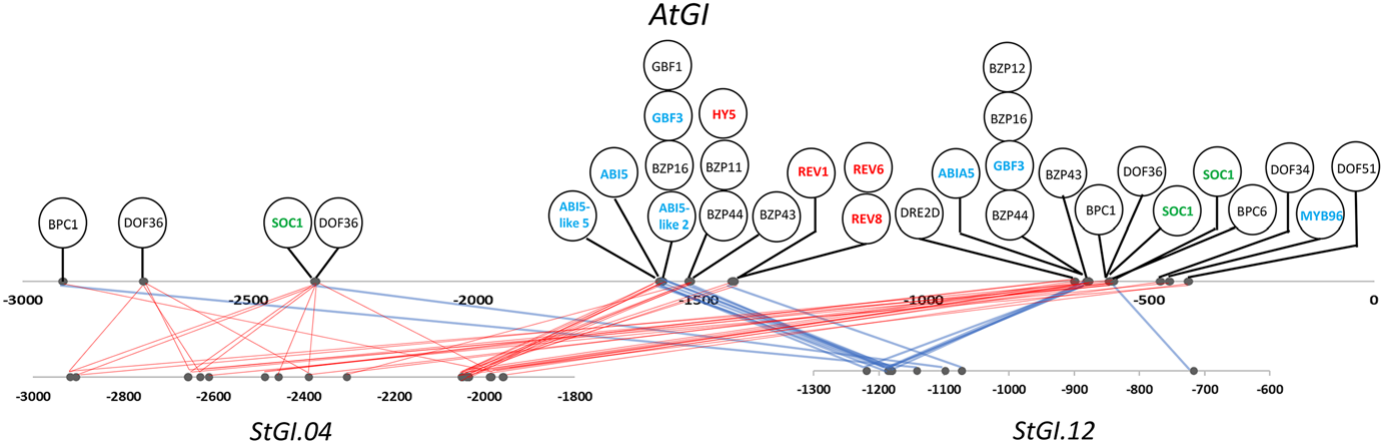
Schematic drawing of TFs binding to both the *Arabidopsis* and *S. tuberosum* Group Phureja *GI* promoters. The name of TFs related to the circadian rhythm, flower development and response to ABA are highlighted in red, green and blue, respectively

Regulation of *GI* gene expression in *Arabidopsis* is well characterized (see Introduction). To see how similar the regulation of *GI* genes is in potato to that of *GI* in *Arabidopsis* the TF binding sites in the − 3-kb sequence of *AtGI* were predicted by PlantRegMap and compared to the binding sites predicted for *StGI* promoters. Using the same parameters as for potato, 160 binding sites of 106 TFs were identified in the *AtGI* promoter region (Table S4). However, several TFs belonged to the same family and recognized overlapping binding sites. All of the TF families found were the same between the *AtGI* and *StGI.04* promoters. Although the NCBI BLASTn comparison did not show significant homology between the *AtGI* and *StGI.04* promoter sequences, TFs regulating similar biological processes and binding to both promoters were recognized (Fig. 3).

In the *AtGI* promoter, binding sites of three different types of TFs (MYB-related, GATA and bZIP) involved in circadian rhythm regulation were detected. The MYB-related TFs were LHY1 and REV1 and/or REV8. LHY1 possessed two binding sites located at approximately − 1.2 and − 1.4 kb. The GATA TF was identified as GAT25, while the bZIP TF was HY5 with binding sites at − 1.3 and − 1.5 kb, respectively. The same type of TF related to the circadian rhythm in the *AtGI* promoter was also identified in *StGI.04*; however, their binding sites were located approximately − 0.5 kb farther from the transcription start site than in *A. thaliana,* and each TF had only a single binding site (Fig. 3). These TFs showed the highest similarity to the *A. thaliana* TFs GATA1, REV1 and HY5. In the *StGI.12* promoter, only the REV1 and/or REV8 binding sites were present at approximately − 1.1 kb upstream of the translation start site.

*AtGI* is involved in flowering time regulation (Rédei 1962). In line with this function, binding sites for TFs affecting flower development were detected in the *AtGI* promoter, i.e., CDF5, TSO1, SOC1-like (AGL20-like) and LHY1. In the *StGI.04* promoter, binding sites for SOC1-like, MYB17, REV8, and CMB1-like were identified, while bHLH130, ATHB51, SOC1, FBP1 and REV8 were predicted to bind to the *StGI.12* promoter. In potato, *GI* indirectly regulates not only flowering time but also tuber initiation (Kloosterman et al. 2013). Corresponding to this function, a binding site for POTH20 (KNOX1) in both potato *GI* promoters, but not in *Arabidopsis*, was found at approximately − 2.0 and − 2.5 kb in *StGI.04* and at approximately − 0.1 kb in the *StGI.12* promoter.

In sum, 20 predicted TFs bound to both *AtGI* and *StGI.04* and 11 TFs common to both *AtGI* and *StGI.12* were found; however, several of them recognized more than one site. Only eight TFs were found in common among all three promoters. These included REV8 with several functions in addition to circadian regulation of gene expression (Singh and Mas 2018), SOC1, a regulator of flower development (Lee et al. 2000), and ABI5 and ABI5-like TFs responding to ABA (Skubacz et al. 2016).

Interestingly, the highest similarity between *AtGI* and *StGI.12* was concentrated at two sites in the *AtGI* promoter, approximately − 0.6 and − 1.0 kb, which were homologous to a short region at approximately − 2.0 kb in the *StGI.04* promoter. The *StGI.12* promoter showed the highest homology to the same *Arabidopsis* regions as *StGI.04*; however, this region was located at − 1.1 to − 1.2 kb in the *StGI.12* promoter. In the case of *StGI.04,* no similarity with the *AtGI* promoter was detected downstream of − 1.8 kb, while in the *StGI.12* promoter, this was the case from − 1.3 kb upstream to the translation start site (Fig. 3). The *StGI-*nonhomologous regions were different in sequence between the two potato *GI* promoters and possessed only five or six putative TF binding sites on each, suggesting that the origin of the differences between the two *GI* promoters of potato are possibly deletions and insertions.

### Identification of the Main *cis*-Acting Regulatory Elements (CAREs) in Potato *GI* Promoters

**Fig. 4:**
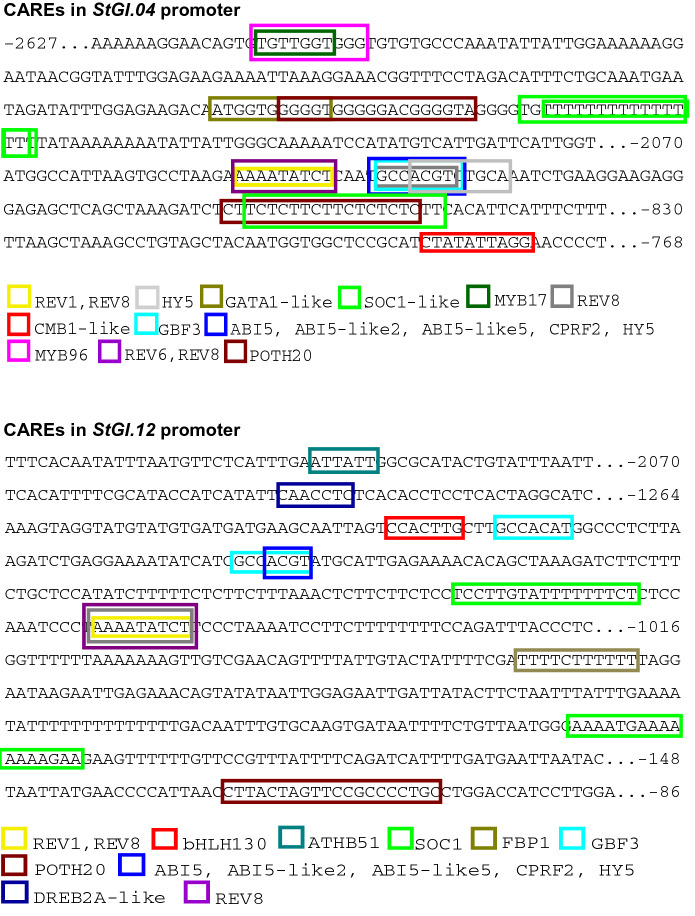
CAREs in *S. tuberosum* Group Phureja *GI* promoters. The colors identify the TFs binding to the promoter elements

The CAREs identified by PlantRegMap in the potato *GI* promoters could be divided into five main categories: circadian rhythm regulation, development, flower development, stress and hormone responses and TFs involved in unknown biological processes. One EVENING ELEMENT (EE) with the consensus sequence AAAATATCT (Harner et al. 2000) in the positive strand starting at − 2051 bp and − 1072 bp was identified in the *StGI.04* and *StGI.12* promoters, respectively, as the binding site of REV1 and/or REV8 related to circadian regulation. REV8, however, is involved not only in circadian regulation but also, among other roles, in the regulation of flower development (Gray et al. 2017) and ABA response (PlantRepMap prediction; Tian et al. 2019). Corresponding to these functions, CAREs with the consensus sequence of GATATTT (GATA-box; Zhou et al. 2008) were found in the negative strand of both *StGI* promoters. The characteristic TTTTTTTTTTTTTTTTT motif of the M-type MADS box TF SOC1-like (PlantRepMap prediction; Tian et al. 2019) and the binding site of ABA-responsive ABI TFs, ACGTG (Choi et al. 2000), also appeared in both *StGI* promoters. The CARE of POTH20 is located at − 1988 bp and − 2478 bp in *StGI.04* and at − 127 bp in *StGI.12*. The locations of these CAREs and others related to circadian rhythm regulation, flower development and ABA response are shown in Fig. 4. A sequence comparison of the CAREs identified in the *StGI* promoters to the corresponding consensus sequences is presented in Tables S5 and S6. Based on PlantRegMap, no ABREL elements are present in the *GI* promoters, but we note that the CACGT motif defined by Berns et al. (2014) as an ABREL core sequence can be found at − 1425, − 2034 and − 2303 bp in *StGI.04* and at − 1179 bp in the *StGI.12* promoter.

**Fig. 5:**
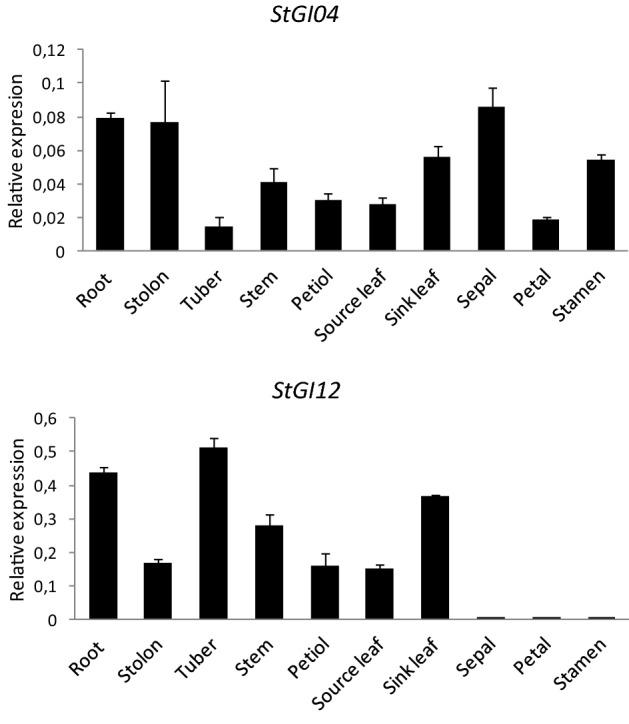
Organ-specific expression of *StGI.04* and *StGI.12* genes in *S. tuberosum* cv. ‘Désirée’. *Y* axis shows mean relative expression values of *StGI* genes compared to the mean expression values of *ACTIN* and *EF1α* ± standard deviation from three technical replicates of one biological replicate composed of a mixture of organs of 3–5 plants. DNA sequences of gene-specific primer pairs used in this study are listed in Table S1

Compared to *S. tuberosum* Group Phureja two insertions were detected in the ‘Désirée’ *StGI.12* promoter region. One of them was a putative binding site for BASIC PENTACYSTEINE-like BBR-BPC family TFs responsive to ethylene and regulating genes involved in development, while the other was for ERF-type TFs involved in the regulation of gene expression by stress factors and by the components of stress signal transduction pathways mediated by ethylene (Fig. 5).

### Organ-Specific Expression of *GI* Genes in Potato

To test the expression of *StGI.04* and *StGI.12* in different organs, potato gene-specific primers were designed (Table S1) and used in RT-qPCR analysis. Root, stolon, tuber, stem, petiole, source- and sink leaf, sepal, petal and stamen samples were collected from greenhouse-grown ‘Désirée’ plants and immediately frozen in liquid nitrogen to isolate RNA for RT-qPCR analysis. *StGI.04* mRNA was detected in each tested organ, with the highest levels in roots, stolons and sepals, the lowest levels in tubers and petals and medium levels in stems, petioles and source and sink leaves. In the case of *StGI.12*, little or no expression was detected in flower organs, whereas it was expressed at relatively high levels in root, tuber and sink leaves and at moderate levels in stolon, stem, petiole and source leaves. In general, the level of *StGI.12* expression was higher than that of *StGI.04*. In roots, for example, the *StGI.12* mRNA level was fivefold higher than that of *StGI.04*, whereas in tubers, it was 30-fold higher. These results indicate that the expression patterns of the two *GI* genes are unique and organ-specific.

### Effect of ABA and Abiotic Stress Treatments on the Expression of *GI* Genes in Potato

**Fig. 6:**
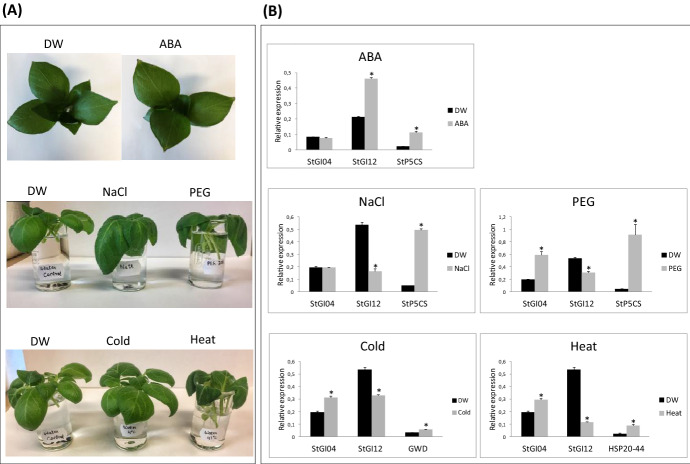
Effects of ABA treatment and abiotic stresses on the detached leaves of *S. tuberosum* cv. ‘Désirée’. **A** Phenotypes of leaves. **B** Relative level of gene expression. The ABA treatment was carried out with 3 source leaves of 6-week-old plants for 24 h under greenhouse conditions. For stress treatments a sample set of 5 source leaves were harvested from 8-week-old plants grown in pots in a greenhouse and subjected to abiotic stresses for 6 h. Efficiency of treatments was tested by the upregulation of *Δ1-PYRROLINE-5-CARBOXYLATE SYNTHETASE* (*StP5CS*), *α-GLUCAN, WATER DIKINASE* (*GWD*) and *HEAT SOCK PROTEIN* *20–44* (*HSP20-44*). *Y* axis shows mean relative expression values of *StGI* genes compared to the mean expression values of *ACTIN* and *EF1α*  ±  standard deviation from three technical replicates of one biological replicate composed of leaves shown in (**A**) part of the figure. Statistical significance of the measurements was determined by Student’s *t*-test (*P*  ≤  0.01) and labeled by an asterisk. DW, distilled water control

In silico analysis of the *GI* promoter regions resulted in the prediction of binding sites for TFs responding to ABA and abiotic stresses, such as salt, water deprivation, cold and heat (Tables S2 and S3). To test the effect of the predicted factors on the transcription of *StGI* genes, detached source leaves of ‘Désirée’ plants grown in a greenhouse were subjected to various treatments and analyzed by RT-qPCR. The stress-inducible genes *P5CS*, *GWD* and *HSP20-44* were used to test the efficiency of the treatments. As shown in Fig. 6a, the leaves became wilted under salt, PEG (an osmotic stressor used to mimic water deprivation) and heat stresses, whereas cold and ABA did not result in phenotypic alterations. PEG, cold and heat upregulated *StGI.04* but downregulated *StGI.12,* and ABA induced *StGI.12* expression but had no effect on *StGI.04* (Fig. 6b). Salt stress repressed *StGI.12* but did not influence the *StGI.04* mRNA level. Thus, one can conclude that the two *StGI* genes respond to abiotic stresses and ABA in different ways.

## Discussion

*GI* is a plant-specific gene involved in multiple biological functions. Terry et al. (2019) demonstrated that the evolution of *GI* occurred through gene duplications resulting in two copies of *GI* in petunia varying in their coding region. Here, we provide evidence that potato also carries two copies of *GI* varying not only in the coding region but also in the promoter region.

*GI* genes show a diurnal cycle of regulation, which was also shown in potato (Morris et al. 2014). Based on the primer sequences presented in that paper, one can conclude that the diurnal expression of *StGI.12*, the copy located on chromosome 12, was detected in that study. We identified one EE as a binding site for REV1/8 related to circadian regulation in both the *StGI.04* and *StGI.12* promoters. Furthermore, ABREL elements, which in combination with EEs are essential to confer a high amplitude diurnal pattern of *GI* expression in *Arabidopsis* (Berns et al. 2014), are present in both *StGI* promoters in the vicinity of the EE motif. Thus, we assume that not only *StGI.12* but also *StGI.04* located on chromosome 4 is under the diurnal cycle of regulation.

GI plays a key role in regulation of the flowering pathway (reviewed by Brandoli et al. 2020). Interestingly, however, unlike *StGI.04*, *StGI.12* is silent in flower organs, although binding sites for TFs involved in the regulation of flower development, including *SOC1*, and pollen maturation, i.e., *ABI5*, were predicted by PlantRegMap not only for *StGI.04* but also for the *StGI.12* promoter. Nevertheless, these TFs may activate *StGI.12*, just not in the mature flowers or stamens tested in our experiment. For example, the floral integrators *SOC1* and *LFY* are expressed in the shoot apical meristem in *Arabidopsis* (Blazquez et al. 1997; Borner et al. 2000).

Organ-specific expression of the *GI* gene was reported in *Arabidopsis,* soybean and sweet potato. In *Arabidopsis*, *AtGI* had high expression levels in inflorescence apices, young flowers, and young siliques (Fowler et al. 1999) and higher mRNA levels in shoots than in roots (Lee and Seo 2018). In soybean (*Glycine max*), three *GI* homologues (*GmGI*) were identified. Under LD conditions, *GmGI* transcripts were expressed at the highest level in the 2^nd^ trifoliolates and floral buds at flowering. Under SD conditions, *GmGI1* showed the highest expression levels in roots at unifoliolate opening and in leaves at flowering. However, *GmGI2* and *GmGI3* always had the highest mRNA levels in roots (Li et al. 2013). Tang et al. (2017) reported that in sweet potato (*Ipomea batata*), *IbGI* expression was stronger in leaves and roots than in stems. In our study, *StGI.04* showed the highest transcript levels in roots, stolons and sepals, while *StGI.12* showed the highest transcript levels in roots, tubers and sink leaves. Thus, the organ specificity of *GI* expression appears to be species- and allele-specific.

In potato, GI plays a key role in tuber initiation (Kloosterman et al. 2013), and POTH20 binding sites were identified in both *StGI* promoters. Rosin et al. (2003) showed that overexpression of *POTH1*, a *KNOTTED*-like homeobox gene with 73% identity to *POTH20*, enhanced in vitro tuberization under both SD and LD photoperiods in several potato lines. If POTH20 can substitute for POTH1, it can be an alternative positive regulator of *StGI* expression.

GI is involved in abiotic stress regulation (reviewed by Jose and Bánfalvi 2019), and in a few plant species, it was shown that the expression of *GI* in leaves is influenced by stresses. The peak level of *AtGI* mRNA, for example, is upregulated under drought stress (Han et al. 2013). Paltiel et al. (2006) demonstrated a strong increase in *GI* expression with increasing temperature in both *Arabidopsis* and *Medicago truncatula*. *IbGI* expression is upregulated by high temperature, drought, and salt stress but downregulated by cold stress (Tang et al. 2017). Here, we showed that the expression of *StGI.04* is induced by cold, heat and osmotic stresses. In contrast, *StGI.12* expression is repressed by the same stresses and by salt stress, which has no effect on *StGI.04*. It has been known for a long time that ABA rapidly accumulates in plants in response to environmental stress and it plays a pivotal role in the reaction to various stimuli (reviewed by Sirko et al. 2021). ABA induced *StGI.12* but not *StGI.04* expression. The reason for all of these differences may be the presence of MYB TF binding sites in *StGI.04*, which are not present in the *StGI.12* promoter. The MYB TFs present in the *StGI.04* promoter respond mainly to salicylic and jasmonic acid, suggesting that different signal transduction pathways lead to up- and downregulation of the two *StGI* genes in response to different abiotic stresses. Nevertheless, the core sequence ACGTG for the binding sites of ABA-responsive TFs is present in both *StGI* promoters.

Comparison of *AtGI* and *StGI*s CAREs resulted in detection of several common elements. Interestingly, similar CAREs in the *AtGI* promoter were located at approximately both − 2.6 kb and − 0.6 kb, suggesting that the evolution of *GI* genes occurred not only by gene duplications, as demonstrated by Terry et al. (2019), but also by duplication of promoter elements.

The expression level of *StGI.12* in root and shoot organs was approximately five times higher and in tubers thirty times higher than the expression level of *StGI.04*. The majority of TF binding sites were found at approximately − 2.0 kb in *StGI.04* and at − 1.2 kb in the *StGI.12* promoter. The regulation of transcription is a complex process that depends on the availability and activity of TFs and the type, number, position and combination of regulatory elements present in and around the promoter (reviewed by Hernandez-Garcia and Finer 2014). Thus, we speculate that the higher activity of *StGI.12* may be explained by the higher proximity of CAREs in the *StGI.12* core promoter region than in the *StGI.04* promoter.

## Supplementary Information

Below is the link to the electronic supplementary material.

Supplementary file1 (PDF 459 kb)

Supplementary file2 (PDF 464 kb)

Supplementary file3 (XLSX 56 kb)

Supplementary file4 (PDF 131 kb)

Supplementary file5 (PDF 205 kb)

## Data Availability

All data generated or analyzed during this study are included in this published article and its supplementary information files.
