# Supplementary material for: In Silico Characterization and Expression Analysis of GIGANTEA Genes in Potato
Source: Biochem Genet. 2022 Mar 11;60(6):2137–54. doi: 10.1007/s10528-022-10214-7 (PMC9617960; doi:10.1007/s10528-022-10214-7)
Supplement: Supplementary file 1 — Supplementary file1 (PDF 459 kb) [file 10528_2022_10214_MOESM1_ESM.pdf]

Biochemical Genetics

In Silico Characterization and Expression Analysis of *GIGANTEA* Genes in Potato

Flóra Karsai-Rektenwald, Khongorzul Odgerel, Jeny Jose[#], Zsófia Bánfalvi[*]

Genetic and Biotechnology Institute, Hungarian University of Agriculture and Life Sciences, 2100 Gödöllő, Szent-Györgyi A. u. 4, Hungary

[*] Corresponding author

E-mail: Banfalvi.Zsofia@uni-mate.hu

***StGI.04* promoter distal**

```
GI04.7      GTGGGTGTGTGCCCAAATATTATTGGAAAAAAGGAATAACGGTATTTGGAGAAGAAAATT   60
GI04.2      GTGGGTGTGTGCCCAAATATTATTGGAAAAAAGGAATAACGGTATTTGGAGAAGAAAATT   60
GI04.6      GTGGGTGTGTGCCCAAATATTATTGGAAAAAAGGAATAACGGTATTTGGAGAAGAAAATT   60
S.ph        GTGGGTGTGTGCCCAAATATTATTGGAAAAAAGGAATAACGGTATTTGGAGAAGAAAATT   60
GI04.1      GTGGGTGTGTGCCCAAATATTATTGGAAAAAAGGAATAACGGTATTTGGAGAAGAAAATT   60
            ************************************************************

GI04.7      AAAGGAAACGGTTTCCTAGACATTTCTGCAAATGAATAGATATTTGGAGAAGACAATGGT   120
GI04.2      AAAGGAAACGGTTTCCTAGACATTTCTGCAAATGAATAGATATTTGGAGAAGACAATGGT   120
GI04.6      AAAGGAAACGGTTTCCTAGACATTTCTGCAAATGAATAGATATTTGGAGAAGACAATGGT   120
S.ph        AAAGGAAACGGTTTCCTAGACATTTCTGCAAATGAATAGATATTTGGAGAAGACAATGGT   120
GI04.1      AAAGGAAACGGTTTCCTAGACATTTCTGCAAATGAATAGATATTTGGAGAAGACAATGGT   120
            ************************************************************

GI04.7      GGGGGTGGGGGACGGGGTAGGGGTGTTTTTTTTTTTTTTTTTTATAAAAAAATATTATTG   180
GI04.2      GGGGGTGGGGGACGGGGTAGGGGTGTTTTTTTTT-TTTTTTTTATAAAAAAATATTATTG   179
GI04.6      GGGGGTGGGGGACGGGGTAGGGGTGTTTTTTTTTTTTTTTTTTATAAAAAAATATTATTG   180
S.ph        GGGGGTGGGGGACGGGGTAGGGGTGTTTTTTTTTTTTTTTTTATAAAAAAAATATTATTG   180
GI04.1      GGGGGTGGGGGACGGGGTAGGGGTGTTTTTTTTTTTTTTTTT ATAAAAAAATATTATTG   179
            ********************************** *******   ***************

GI04.7      GGCAAAAATCCATATGTCATTGATTCATTGGTTACTTTTTATTTTTATTCAAAAATAATT   240
GI04.2      GGCAAAAATCCATATGTCATTGATTCATTGGTTACTTTTTATTTTTATTCAAAAATAATT   239
GI04.6      GGCAAAAATCCATATGTCATTGATTCATTGGTTACTTTTTATTTTTATTCAAAAATAATT   240
S.ph        GGCAAAAATCCATATGTCATTGATTCATTGGTTACTTTTTATTTTTATTCAAAAATAATT   240
GI04.1      GGCAAAAATCCATATGTCATTGATTCATTGGTTACTTTTTATTTTTATTCAAAAATAATT   239
            ************************************************************

GI04.7      ATTTAAGAATTGTTTTTGATGCAAATGACAAATGTCGTCGTTTAATTTGTCACTTTACAC   300
GI04.2      ATTTAAGAATTGTTTTTGATGCAAATGACAAATGTCGTCGTTTAATTTGTCACTTTACAC   299
GI04.6      ATTTAAGAATTGTTTTTGATGCAAATGACAAATGTCGTCGTTTAATTTGTCACTTTACAC   300
S.ph        ATTTAAGAATTGTTTTTGATGCAAATGACAAATGTCGTCGTTTAATTTGTCACTTTACAC   300
GI04.1      ATTTAAGAATTGTTTTTGATGCAAATGACAAATGTCGTCGTTTAATTTGTCACTTTACAC   299
            ************************************************************

GI04.7      GTCATTCGCGAGTGTAACACACACCCTATATATTTTTGTTGATTGGTAAAAGAGTGTCAA   360
GI04.2      GTCATTCGCGAGTGTAACACACACCTTATATATTTTTGTTGATTGGTAAAAGAGTGTCAA   359
GI04.6      GTCATTCGCGAGTGTAACACACACCTTATATATTTTTGTTGATTGGTAAAAGAGTGTCAA   360
S.ph        GTCATTCGCGAGTGTAACACACACCTTATATATTTTTGTTGATTGGTAAAAGAGTGTCAA   360
GI04.1      GTCATTCGCGAGTGTAACACACACCTTATATATTTTTGTTGATTGGTAAAAGAGTGTCAA   359
            ************************ ***********************************

GI04.7      AATGACACATTAAGACTCTACATGAAGTGTCTAAAATAAATATCGTCCGGTTAAGATGTC   420
GI04.2      AATGACACATTAAGACTCTACATGAAGTGTCTAAAATAAATATCGTCCGGTTAAGATGTC   419
GI04.6      AATGACACATTAAGACTCTACATGAAGTGTCTAAAATAAATATCGTCCGGTTAAGATGTC   420
S.ph        AATGACACATTAAGACTCTACATGAAGTGTCTAAAATAAATATCGTCCGGTTAAGATGTC   420
GI04.1      AATGACACATTAAGACTCTACATGAAGTGTCTAAAATAAATATCGTCCGGTTAAGATGTC   419
            ************************************************************

GI04.7      TAAGTGAAACTTCGTGTCAACTTTAAGGGGCCACTGATGGGTTAGACCTATGGGTGTTTG   480
GI04.2      TAAGTGAAACTTCGTGTCAACTTTAAGGGGCCACTGATGGGCTAGACCTATGGGTGTTTG   479
GI04.6      TAAGTGAAACTTCGTGTCAACTTTAAGGGGCCACTGATGGGTTAGACCTATGGGTGTTTG   480
S.ph        TAAGTGAAACTTCGTGTCAACTTTAAGGGGCCACTGATGGGTTAGACCTATGGGTGTTTG   480
GI04.1      TAAGTGAAACTTCGTGTCAACTTTAAGGGGCCACTGATGGGTTAGACCTATGGGTGTTTG   479
            ***************************************** ******************

GI04.7      TCATGGGTGCATGTCATGATGATTCATGATGGTGTTGAATCATATGACACATGGCCATTA   540
GI04.2      TCATGGGTGCATGTCATGACGATTCATGGTGGTGTTGAATCATATGACACATGGCCATTA   539
GI04.6      TCATGGGTGCATGTCATGATGATTCATGATGGTGTTGAATCATATGACACATGGCCATTA   540
S.ph        TCATGGGTGCATGTCATGATGATTCATGATGGTGTTGAATCATATGACACATGGCCATTA   540
GI04.1      TCATGGGTGCATGTCATGATGATTCACGATGGTGTTGAATCATATGACACATGGCCATTA   539
            ******************* ****** * *******************************

GI04.7      AGTGCCTAAGAAAAATATCTCAATGCCACGTCTGCAAATCTGAAGGAAGAGGGAGAGCTC   600
GI04.2      AGTGCCTAAGAAAAATATCTCAATGCCACGTCTGCAAATCTGAAGGAAGAGGGAGAGCTC   599
GI04.6      AGTGCCTAAGAAAAATATCTCAATGCCACGTCTGCAAATCTGAAGGAAGAGGGAGAGCTC   600
```

```
S.ph        AGTGCCTAAGAAAAAATATCTCAATGCCACGTCTGCAAATCTGAAGGAAGAGGGAGAGCTC    600
GI04.1      AGTGCCTAAGAAAAAATATCTCAATGCCACGTCTGCAAATCTGAAGGAAGAGGGAGAGCTC    599
            ************************************************************

GI04.7      AGCTAAAGATCTCTTCTCTTCTTCTCTTTCTTCACATTCATTTCTTTTCTCTCTACTTTT    660
GI04.2      AGCTAAAGATCTCTTCTCTTCTTCTCTCTCTTCACATTCATTTCTTTTCTCTCTACTTTT    659
GI04.6      AGCTAAAGATCTCTTCTCTTCTTCTCTCTCTTCACATTCATTTCTTTTCTCTCTACTTTT    660
S.ph        AGCTAAAGATCTCTTCTCTTCTTCTCTCTCTTCACATTCATTTCTTTTCTCTCTACTTTT    660
GI04.1      AGCTAAAGATCTCTTCTCTTCTTCTCTCTCTTCACATTCATTTCTTTTCTCTCTACTTTT    659
            *************************** ********************************

GI04.7      TTCCTCCAACCAAAACCTGTTAAACAATCTTCTTCAAATTTCACCTTCCAATTTCAGGTA    720
GI04.2      TTCCTCCAACCAAAACCTGTTAAACAATCTTCTTCAAATTTCACCTTCCAATTTCAGGTA    719
GI04.6      TTCCTCCAACCAAAACCTGTTAAACAATCTTCTTCAAATTTCACCTTCCAATTTCAGGTA    720
S.ph        TTCCTCCAACCAAAACCTGTTAAACAATCTTCTTCAAATTTCACCTTCCAATTTCAGGTA    720
GI04.1      TTCCTCCAACCAAAACCTGTTAAACAATCTTCTTCAAATTTCACCTTCCAATTTCAGGTA    719
            ************************************************************

GI04.7      CCATTTTTTTCTTTAATTTTCTTCTATAACCTCTGAATTTTTTTCTAGTTGTTTAAAGCT    780
GI04.2      CCATTTTTTTCTTTAATTTTCTTCTATAAC-TCTGAATTTTTTTCTAGTTGTTTAAAGCT    778
GI04.6      CCATTTTTTTCTTTAATTTTCTTCTATAAC-TCTGAATTTTTTTCTAGTTGTTTTAAAGC    779
S.ph        CCATTTTTTTCTTTAATTTTCTTCTATAAC-TCTGAATTTTTTTCTAGTTGTTTAAAGCT    779
GI04.1      CCATTTTTTTCTTTAATTTTCTTCTATAAC-TCTGAATTTTTTTCTAGTTGTTTAAAGCT    778
            ****************************** *********************** **

GI04.7      CCGTTTTCTCTA-TGGGATTTGTAATTAATATCTTCNTGCTGTTGTTGATTTGNNATAAC    839
GI04.2      -CCGTTTCTCTA-TGGGATTTGTAATTAATATCTC--TGCTGTTGTTGATTTGTTATAAC    834
GI04.6      TCCGTTTCTCTA-TGGGATTTGTAATTAATATCTC--TGCTGTTGTTGATTTGTTATAAC    837
S.ph        -CCGTTTCTCTA-TGGGATTTGTAATTAATATCTC--TGCTGTTGTTGATTTGTTATAAC    835
GI04.1      -CCGTTTCTCTA-TGGGATTTGTAATTAATATCTC--TGCTGTTGTTGATTTGTTATAAC    834
             *  ******** *********************   ****************  *****

GI04.7      TTTTCTTGCCTTTTGCCTCAGTTCATAATGAAAATTNGNTTAGGAAAGTATGGAGATTCG    899
GI04.2      TT-TCTTGC-TTTTGCCTCAGTTCATAATGAAA-TTGGTT-AGGAA-GTATGAGA-TTCG    887
GI04.6      TT-TCTTGC-TTTTGCCTCAGTTCATAATGAAA-TTGGNTTAGGGAAGTATGGAGATTCG    894
S.ph        TT-TCTTGC-TTTTGCCTCAGTTCATAATGAAA-TTGGTT-AGGAA-GTATGGAGATTCG    890
GI04.1      TT-TCTTGC-TTTTGCCTCAGTTCATAATGAAA-TTGGTT-AGGAA-GTATGGAGATTCG    889
            ** ****** *********************** ** * * *** * *****    ****

GI04.7      CTTTTTGTTGATCAAGAGAAATATATGTTGTTAATAAGGTTATTACAAATATTTATTGGA    959
GI04.2      CTTTTTN-TGATCAAGAGAAATATATNT-GTTAATAAGGTTATTACAAATATTTATTGGA    947
GI04.6      CTTTTTGTTGATCAAGAGAAATATATGTTGTTAATAAGGTTATTACAAATATTTATTGGA    954
S.ph        CTTTTTGTTGATCAAGAGAAATATATGTTGTTAATAAGGTTATTACAAATATTTATTGGA    950
GI04.1      CTTTTTGTTGATCAAGAGAAATATATGTTGTTAATAAGGTTATTACAAATATTTATTGGA    949
            ******  ****************** * *******************************

GI04.7      ACTAAAATGTTATTCCA-GTAATTTGGAATTTATAGAATTGTTGAGTTTTACCAAACATT    1018
GI04.2      ACTAAAATGTTATTNCCAGTAATTTGGAATTTATAGAATTGTTGAGTTTTACCAAAC-AT    1006
GI04.6      ACTAAAATGTTATTCCA-GTAATTTGGAATTTATAGAATTGTTGAGTTTTACCAAACATT    1013
S.ph        ACTAAAATGTTATTCCA-GTAATTTGGAATTTATAGAATTGTTGAGTTTTACCAAACATT    1009
GI04.1      ACTAAAATGTTATTCCA-GTAATTTGGAATTTATAGAATTGTTGAGTTTTACCAAACATT    1008
            ************** *  ***************************************  *

GI04.7      TTTTTTTCGGATTTCCACTTGGTGTCTTGTACTCACATTGGAAGCCGACTAAATCCGTAG    1078
GI04.2      TTTTTTTTGGATTTCCACTTGGTGTCTTGTACTCACATTGGAAGCCGACTAAATCCGTAG    1066
GI04.6      TTTTTTTTGGATTTCCACTTGGTGTCTTGTACTCACATTGGAAGCCGACTAAATCCGTAG    1073
S.ph        TT-TTTTTGGATTTCCACTTGGTGTCTTGTACTCACATTGGAAGCCGACTAAATCCGTAG    1068
GI04.1      TT-TTTTTGGATTTCCACTTGGTGTCTTGTACTCACATTGGAAGCCGACTAAATCCGTAG    1067
            ** **** ****************************************************

GI04.7      GTTCCATTTTTTGGGGAGACTCTGCCAAAAGGTTTTTTTCTATTCCTGAGGGTTCGAATA    1138
GI04.2      GTTCCATTTTTTGGGGAGACTCTGCCAAAAGGTTTTTTTCTATTCCTGAGGGTTCGAATA    1126
GI04.6      GTTCCATTTTTTGGGGAGACTCTGCCAAAGGGTTTTTTTCTATTCCTGAGGGTTCGAATA    1133
S.ph        GTTCCATTTTTTGGGGAGACTCTGCCAAAAGGTTTTTTTCTATTCCTGAGGGTTCGAATA    1128
GI04.1      GTTCCATTTTTTGGGGAGACTCTGCCAAAAGGTTTTTTTCTATTCCTGAGGGTTCGAATA    1127
            ***************************** ******************************

GI04.7      CAAAAGTCTTATTAAGGGTGGAGGGATCTCAACCATGCCACACAATTCACGTTGCTAGTT    1198
```

```
GI04.2      CAAAAGTCTTATTAAGGGTGGAGGGATCTCAACCATGCCACACAATTCACGTTGCTAGTT    1186
GI04.6      CAAAAGTCTTATTAAGGGTGGAGGGATCTCAACCATGCCACACAATTCACGTTGCTAGTT    1193
S.ph        CAAAAGTCTTATTAAGGGTGGAGGGATCTCAACCATGCCACACAATTCACGTTGCTAGTT    1188
GI04.1      CAAAAGTCTTATTAAGGGTGGAGGGATCTCAACCATGCCACACAATTCACGTTGCTAGTT    1187
            ************************************************************

GI04.7      TTACAGTTGAATAGATTCTGTCCATTGTATTCCTTATGCCTATTTATACATATGTTTTTT    1258
GI04.2      TTACAGTTGAATAGATTCTGTCCATTGTATTCCTTATGCCTATTTATACATATGTTTTTT    1246
GI04.6      TTACAGTTGAATAGATTCTGTCCATTGTATTCCTTATGCCTATTTATACATATGTTTTTT    1253
S.ph        TTACAGTTGAATAGATTCTGTCCATTGTATTCCTTATGCCTATTTATACATATGTTTTTT    1248
GI04.1      TTACAGTTGAATAGATTCTGTCCATTGTATTCCTTATGCCTATTTATACATATGTTTTTT    1247
            ************************************************************

GI04.7      TACTTGCTAATGTTTTCCTAGATTGATTTTCATACTGTCCAAGTTTACTTTGAATATAGC    1318
GI04.2      TACTTGCTAATGTTTTCCTAGATTGATTTTCATACTGTCCAAGTTTACTTTGAATATAGC    1306
GI04.6      TACTTGCTAATGTTTTCCCAGATTGATTTTCATACTGTCCAAGTTTACTTTGAATATAGC    1313
S.ph        TACTTGCTAATGTTTTCCTAGATTGATTTTCATACTGTCCAAGTTTACTTTGAATATAGC    1308
GI04.1      TACTTGCTAATGTTTTCCTAGATTGATTTTCATACTGTCCAAGTTTACTTTGAATATAGC    1307
            ****************** *****************************************

GI04.7      TCGTTATGGACTGGTGCTTACTTGAAAACTCTTAGTATATTAGTTAAAGATATAACCACT    1378
GI04.2      TCGTTATGGACTGGTGCTTACTTGAAAACTCTTAGTATATTAGTTAAAGATATAACCACT    1366
GI04.6      TCGTTATGGACTGGTGCTTACTTGAAAACTCTTAGTATATTAGTTAAAGATATAACCACT    1373
S.ph        TCGTTATGGACTGGTGCTTACTTGAAAACTCTTAGTATATTAGTTAAAGATATAACCACT    1368
GI04.1      TCGTTATGGACTGGTGCTTACTTGAAAACTCTTAGTATATTAGTTAAAGATATAACCACT    1367
            ************************************************************

GI04.7      AGAGTATTATTAGAGCAAAGGCGGAGTCGAGGATTTTTAGTTTATAGATTTTGAATCACA    1438
GI04.2      AGAGTATTATTAGAGCAAAGGCGGAGTCGAGGATTTTTAGTTTATAGATTTTGAATCACA    1426
GI04.6      AGAGTATTATTAGAGCAAAGGCGGAGTCGAGGATTTTTAGTTTATAGATTTTGAATCACA    1433
S.ph        AGAGTATTATTAGAGCAAAGGCGGAGTCGAGGATTTTTAGTTTATAGATTTTGAATCACA    1428
GI04.1      AGAGTATTATTAGAGCAAAGGCGGAGTCGAGGATTTTTAGTTTATAGATTTTGAATCACA    1427
            ************************************************************

GI04.7      ATCCATTTTTTT-TTTTTACCCTCCCTAGGAGCTCCCACCCCTTTTGCTCCCTTGGTGAC    1497
GI04.2      ATCCATTTTT---TTTTTACCCTCCCTAGGAGCTCCCACCCCTTTTGCTCCCTTGGTGAC    1483
GI04.6      ATCCATTTTTTTTTTTTTACCCTCCCTAGGAGCTCCCACCCCTTTTGCTCCCTTGGTGAC    1493
S.ph        ATCCATTTTTTT-TTTTTACCCTCCCTAGGAGCTCCCACCCCTTTTGCTCCCTTGGTGAC    1487
GI04.1      ATCCATTTTTT--TTTTTACCCTCCCTAGGAGCTCCCACCCCTTTTGCTCCCTTGGTGAC    1485
            **********   ***********************************************

GI04.7      TCGAACTCGCAACCTTCGGGTTGGAAGTGAGGGGTGCTTACCATCCGAGCAACTCCCTCT    1557
GI04.2      TCGAACTCGCAACCTTCGGGTTGGAAGTGAGGGGTGCTTACCATCCGAGCATCTCCCTCT    1543
GI04.6      TCGAACTCGCTACCTTCGGGTTGGAAGTGTGGGGTGCTTACCATCCGAGCAACTCCCTCT    1553
S.ph        TCGAACTCGCAACCTTCGGGTTGGAAGTGAGGGGTGCTTACCATCCGAGCAACTCCCTCT    1547
GI04.1      TCGAACTCGCAACCTTCGGGTTGGAAGTGAGGGGTGCTTACCATCCGAGCAACTCCCTCT    1545
            ********** ****************** ********************* ********

GI04.7      TGTCACAATCCATTTTGCTTACTGGGTTTTGGATACATTATTTTTTTTGATCAGATAAATG    1617
GI04.2      TGTCACAATCCATTTTGCTTACTGGGTTTTGGATACATTATTTTTTTTGATCAGATAAATG    1603
GI04.6      TGTCACAATCCATTTTGCTTACTGGGTTTTGGATACATTACTTTTTTTGATCAGATAAATG    1613
S.ph        TGTCACAATCCATTTTGCTTACTGGGTTTTGGATACATTATTTTTTTTGATCAGATAAATG    1607
GI04.1      TGTCACAATCCATTTTGCTTACTGGGTTTTGGATACATTATTTTTTTTGATCAGATAAATG    1605
            **************************************** *******************

GI04.7      ATTTTATAAATAAAGGGGCAAAGCCGTATACCATAAGTAGAGAACCCACAACAAAATATG    1677
GI04.2      ATTTTATAAATAAAGGGGCAAAGCCGTATACCATAAGTAGAGAACCCACAACAAAATATG    1663
GI04.6      ATTTTATAAATAAAGGGGCAAAGCCGTATACCATAAGTAGAGAACCCACAACAAAATATG    1673
S.ph        ATTTTATAAATAAAGGGGCAAAGCCGTATACCATAAGTAGAGAACCCACAACAAAATATG    1667
GI04.1      ATTTTATAAATAAAGGGGCAAAGCCGTATACCATAAGTAGAGAACCCACAACAAAATATG    1665
            ************************************************************

GI04.7      TTTCTCTATGAATGAAACCAATCATCTGTAGGAACACCAAACATAAACTAAAAACAAAAG    1737
GI04.2      TTTCTCTATGAATGAAACCAATCATCTGTAGGAACACCAAACATAAACTAAAAACAAAAG    1723
GI04.6      TTTCTCTATGAATGAAACCAATCATCTGTAGGAACACCAAACATAAACTAAAAACAAAAG    1733
S.ph        TTTCTCTATGAATGAAACCAATCATCTGTAGGAACACCAAACATAAACTAAAAACAAAAG    1727
GI04.1      TTTCTCTATGAATGAAACCAATCATCTGTAGGAACACCAAACATAAACTAAAAACAAAAG    1725
            ************************************************************
```

```
GI04.7      GTTTGCAGCTTTACATATTATTTAAACATATGAAATACAAGGTTTAAGCTAAAGCCTGTA   1797
GI04.2      GTTTGCAGCTTTACATATTATTTAAACATATGAAATACAAGGTTTAAGCTAAAGCCTGTA   1783
GI04.6      GTTTGCAGCTTTACATATTATTTAAACATATGAAATACAAGGTTTAAGCTAAAGCCTGTA   1793
S.ph        GTTTGCAGCTTTACATATTATTTAAACATATGAAATACAAGGTTTAAGCTAAAGCCTGTA   1787
GI04.1      GTTTGCAGCTTTACATATTATTTAAACATATGAAATACAAGGTTTAAGCTAAAGCCTGTA   1785
            ************************************************************

GI04.7      GCTACAATGGTGGCTCCG   1815
GI04.2      GCTACAATGGTGGCTCCG   1801
GI04.6      GCTACAATGGTGGCTCCG   1811
S.ph        GCTACAATGGTGGCTCCG   1805
GI04.1      GCTACAATGGTGGCTCCG   1803
            ******************
```

***StG.04* promoter proximal**

```
GI04.6      TAGCTACAATGGTGGCTCCGCATCTATATTAGGAACCCCTAGTGTTAGATAATCCATAAT   60
GI04.2      TAGCTACAATGGTGGCTCCGCATCTATATTAGGAACCCCTAGTGTTAGATAATCCATAAT   60
GI04.9      TAGCTACAATGGTGGCTCCGCATCTATATTAGGAACCCCTAGTGTTAGATAATCCATAAT   60
GI04.11     TAGCTACAATGGTGGCTCCGCATCTATATTAGGAACCCCTAGTGTTAGATAATCCATAAT   60
S.ph        TAGCTACAATGGTGGCTCCGCATCTATATTAGGAACCCCTAGTGTTAGATAATCCATAAT   60
            ************************************************************

GI04.6      TATGGAGTATTGAAATAATACGGACCCAAGCAAAATATATTTATGATATTGAGGATTCAT   120
GI04.2      TATGGAGTATTGAAATAATACGGACCCAAGCAAAATATATTTATGATATTGAGGATTCAT   120
GI04.9      TATGGAGTATTGAAATAATACGGACCCAAGCAAAATATATTTATGATATTGAGGATTCAT   120
GI04.11     TATGGAGTATTGAAATAATACGGACCCAAGCAAAATATATTTATGATATTGAGGATTCAT   120
S.ph        TATGGAGTATTGAAATAATACGGACCCAAGCAAAATATATTTATGATATTGAGGATTCAT   120
            ************************************************************

GI04.6      ACATCCAACCCAACTAGTTTGGAATTGAGGCGTAGTTGTTGTATAATTCTTTGTTGAGCT   180
GI04.2      ACATCCAACCCAACTAGTTTGGAATTGAGGCGTAGTTGTTGTATAATTCTTTGTTGAGCT   180
GI04.9      ACATCCAACCCAACTAGTTTGGAATTGAGGCGTAGTTGTTGTATAATTCTTTGTTGAGCT   180
GI04.11     ACATCCAACCCAACTAGTTTGGAATTGAGGCGTAGTTGTTGTATAATTCTTTGTTGAGCT   180
S.ph        ACATCCAACCCAACTAGTTTGGAATTGAGGCGTAGTTGTTGTATAATTCTTTGTTGAGCT   180
            ************************************************************

GI04.6      TGAAAATCAATTTTCCTTGTAGTCATATGATTTTTGTTTGTGCCAATTTAATGGGCTGTT   240
GI04.2      TGAAAATCAATTTTCCTTGTAGTCATATGATTTTTGTTTGTGCCAATTTAATGGGCTGTT   240
GI04.9      TGAAAATCAATTTTCCTTGTAGTCATATGATTTTTGTTTGTGCCAATTTAATGGGCTGTT   240
GI04.11     TGAAAATCAATTTTCCTTGTAGTCATATGATTTTTGTTTGTGCCAATTTAATGGGCTGTT   240
S.ph        TGAAAATCAATTTTCCTTGTAGTCATATGATTTTTGTTTGTGCCAATTTAATGGGCTGTT   240
            ************************************************************

GI04.6      AAGTGAATTACCATATTCCCCTATTTTGCCAGTTTCATCATCACTTCTCTCTGATGTTTT   300
GI04.2      AAGTGAATTACCATATTCCCCTATTTTGCCAGTTTCATCATCATTTCTCTCTGATGTTTT   300
GI04.9      AAGTGAATTACCATATTCCCCTATTTTGCCAGTTTCATCATCATTTCTCTCTGATGTTTT   300
GI04.11     AAGTGAATTACCATATTCCCCTATTTTGCCAGTTTCATCATCATTTCTCTCTGATGTTTT   300
S.ph        AAGTGAATTACCATATTCCCCTATTTTGCCAGTTTCATCATCATTTCTCTCTGATGTTTT   300
            ******************************************* ****************

GI04.6      AATTTTCATAGAGTTTTGGTTTTATATTTCGCTGTGTCAGCAAGGATGCCGTCATTGAAT   360
GI04.2      AATTTTCATAGAGTTTTGGTTTTATATTTCGCTGTGTCAGCAGGGATGCCGTCATTGAAT   360
GI04.9      AATTTTCATAGAGTTTTGGTTTTATATTTCGCTGTGTCAGCAAGGATGCCGTCATTGAAT   360
GI04.11     AATTTTCATAGAGTTTTGGTTTTATATTTCGCTGTGTCAGCAAGGATGCCGTCATTGAAT   360
S.ph        AATTTTCATAGAGTTTTGGTTTTATATTTCGCTGTGTCAGCAAGGATGCCGTCATTGAAT   360
            ***************************************** ******************

GI04.6      GAAAAACTTGATATATGAAACAGCGTGTTTTCCCTCTAGATTTCCTATGGAAATCCTCCA   420
GI04.2      GAAAAACTTGATATATGAAACAGCGTGTTTTCCCTCTAGATTTCCTATGGAAATCCTCCA   420
GI04.9      GAAAAACTTGATATATGAAACAGCGTGTTTTCCCTCTAGATTTCCTATGGAAATCCTCCA   420
GI04.11     GAAAAACTTGATATATGAAACAGCGTGTTTTCCCTCTAGATTTCCTATGGAAATCCTCCA   420
S.ph        GAAAAACTTGATATATGAAACAGCGTGTTTTCCCTCTAGATTTCCTATGGAAATCCTCCA   420
            ************************************************************
```

```
GI04.6      ATACTTCTTTCACACAACTTTTGGTGCTTGGTTTGATCAAACTAATTAACCTTCTTTTTT   480
GI04.2      ATACTTCTTTCACACAACTTTTGGTGCTTGGTTTGATCAAACTAATTAACCTTCTTTTTT   480
GI04.9      ATACTTCTTTCACACAACTTTTGGTGCTTGGTTTGATCAAACTAATTAACCTTCTTTTTT   480
GI04.11     ATACTTCTTTCACACAACTTTTGGTGCTTGGTTTGATCAAACTAATTAACCTTCTTTTTT   480
S.ph        ATACTTCTTTCACACAACTTTTGGTGCTTGGTTTGATCAAACTAATTAACCTTCTTTTTT   480
            ************************************************************

GI04.6      TATCTCTTGGAGGATGCTTATTTCTTTATGCATCTGTCTAATAGTTTTGACTATCATTGT   540
GI04.2      TATCTCTTGGAGGATGCTTATTTCTTTATGCATCTGTCTAATAGTTTTGACTATCATTGT   540
GI04.9      TATCTCTTGGAGGATGCTTATTTCTTTATGCATCTGTCTAATAGTTTTGACTATCATTGT   540
GI04.11     TATCTCTTGGAGGATGCTTATTTCTTTATGCATCTGTCTAATAGTTTTGACTATCATTGT   540
S.ph        TATCTCTTGGAGGATGCTTATTTCTTTATGCATCTGTCTAATAGTTTTGACTATCATTGT   540
            ************************************************************

GI04.6      GTTTTTCTGTTTTGCAGGACAAATAGTCATATGTTGTAGTTAGTATCTGCTTACTATTTA   600
GI04.2      GTTTTTCTGTTTTGCAGGACAAATAGTCATATGTTGTAGTTAGTATCTGCTTACTATTTA   600
GI04.9      GTTTTTCTGTTTTGCAGGACAAATAGTCATATGTTGTAGTTAGTATCTGCTTACTATTTA   600
GI04.11     GTTTTTCTGTTTTGCAGGACAAATAGTCATATGTTGTAGTTAGTATCTGCTTACTATTTA   600
S.ph        GTTTTTCTGTTTTGCAGGACAAATAGTCATATGTTGTAGTTAGTATCTGCTTACTATTTA   600
            ************************************************************

GI04.6      ATCAGGGAATTTACATTCCCGTAACCTGCATATTATTTTCAGAGATACCTTTGAAAGCTC   660
GI04.2      ATCAGGGAATTTACATTCCCGTAACCTGCATATTATTT-CAGAGATACCTT-GAA-GCTC   657
GI04.9      ATCAGGGAATTTACATTCCCGTAACCTGCATATTATTT-CAGAGATACCTT-GAA-GCTC   657
GI04.11     ATCAGGGAATTTACATTCCCGTAACCTGCATATTATTT-CAGAGATACCTT-GAA-GCTC   657
S.ph        ATCAGGGAATTTACATTCCCGTAACCTGCATATTATTT-CAGAGATACCTT-GAA-GCTC   657
            ************************************** ************ *** ****

GI04.6      TGAGGTGGTTTTGCTCGTTTAGAAGTTTCTGGACCATCCTTTGCTAGGATTCCTAGATGT   720
GI04.2      TGAGGTGGTTTTGCTCGT-TAGAAGTT--TGGACCATCCTTTGCTAGGATCCTA-GATGT   714
GI04.9      TGAGGTGGTTTTGCTCGT-TAGAAGTT-CTGGACCATCCTTTGCTAGGATCCTA-GATGT   714
GI04.11     TGAGGTGGTTTTGCTCGT---GAAGTT-CTGGACCATCCTTTGCTAGGATCCTA-GATGT   714
S.ph        TGAGGTGGTTTTGCTCGT-TAGAAGTT-CTGGACCATCCTTTGCTAGGATCCTA-GATGT   714
            ******************   ******  ***********************   *****

GI04.6      TGGGAATTTACAATCTTGTGGATCCTAGATTGGAAGTGGTACTTTGATGGGCCTAATTTT   780
GI04.2      TG---AAATTACAATCTGTGGATCCTAGATTGGAAGTGGTACTT-GATGG-CCTAATTT-   770
GI04.9      TG---AAATTACAATCTGTGGATCCTAGATTGGAAGTGGTACTT-GATGG-CCTAATTT-   770
GI04.11     TG---AAATTACAATCTGTGGATCCTAGATTGGAAGTGGTACTT-GATGG-CCTAATTT-   770
S.ph        TG---AAATTACAATCTGTGGATCCTAGATTGGAAGTGGTACTT-GATGG-CCTAATTT-   770
            **   ** * *** * **************************** ***** ********

GI04.6      GTAGCATCCCTCTCATTATTGATTAAAATTCTTGGGATTGATAGCAGTTTT   831
GI04.2      GTAGCATCC--CTCAT-ATTGAT-AAAATTCTTGG-ATTGATAGCAGTTAT   815
GI04.9      GTAGCATCC-TCTCAT-ATTGAT-AAAATTCTTGG-ATTGATAGCAGTTAT   815
GI04.11     GTAGCATCC-TCTCAT-ATTGAT-AAAATTCTTGG-ATTGATAGCAGTTAT   815
S.ph        GTAGCATCC-TCTCAT-ATTGAT-AAAATTCTTGG-ATTGATAGCAGTTAT   815
            *********  ***** ****** *********** ************* *
```

Fig. S1 Multiple sequence alignment of *StGI.04* promoter fragments cloned from *S. tuberosum* cv. 'Désirée' and retrieved for *S. tuberosum* Group Phureja (*S.ph*) from SpudDB. *StG.04* fragments extending from -2601 to -816 bp and from -816 to +65 bp were PCR amplified from the genomic DNA of 'Désirée' with the primer pairs StGI.04 -2601 FW and StGI.04 -816 R, and StG.04 -816 FW and StGI.04 +65 R, respectively. Primer sequences are presented in Table S1. The fragments were cloned into pGEM-T-Easy and DNA of four recombinant clones was Sanger sequenced. The program Clustal Omega was used for multiple sequence alignment.
