# Supplementary material for: In Silico Characterization and Expression Analysis of GIGANTEA Genes in Potato
Source: Biochem Genet. 2022 Mar 11;60(6):2137–54. doi: 10.1007/s10528-022-10214-7 (PMC9617960; doi:10.1007/s10528-022-10214-7)
Supplement: Supplementary file 2 — Supplementary file2 (PDF 464 kb) [file 10528_2022_10214_MOESM2_ESM.pdf]

Biochemical Genetics

# *In Silico* Characterization and Expression Analysis of *GIGANTEA* Genes in Potato

**Flóra Karsai-Rektenwald, Khongorzul Odgerel, Jeny Jose[#], Zsófia Bánfalvi[*]**

Genetic and Biotechnology Institute, Hungarian University of Agriculture and Life Sciences, 2100 Gödöllő, Szent-Györgyi A. u. 4, Hungary

[*] Corresponding author

E-mail: Banfalvi.Zsofia@uni-mate.hu

### *StGI.12* promoter distal

```
S.ph        TTAGACAGCTCCTCCCACTAATGCATTTTCTATTCAATT---------------------   39
GI12.2      TTAGACAGCTCCTCCCACTAATGCATTTTCTATTCAATTAT-------------------   41
GI12.9      TTAGACAGCTCCTCCCACTAATGCATTTTCTATTCAATTATTCACCCAATTAAATAATTT   60
GI12.1      TTAGACAGCTCCTCCCACTAATGCATTTTCTATTCAATTAT-------------------   41
GI12.10     TTAGACAGCTCCTCCCACTAATGCATTTTCTATTCAATTATTCACCCAATTAAATAATTT   60
            ***************************************

S.ph        ------------------------ATTCACCCAATTAAATAATTTTTAATTTCACTTCTT   75
GI12.2      ------------------------ATTCACCCAACTAAATAATTTTTAATTTCACTTCTT   77
GI12.9      TTAATTTCACTTCTTCAAGTTTTTGTTCACCCAATTAAATAATTTTTAATTTCACTTCTT   120
GI12.1      ------------------------ATTCACCCAATTAAATAATTTTTAATTTCACTTCTT   77
GI12.10     TTAATTTCACTTCTTCAAGTTTTTGTTCACCCAATTAAATAATTTTTAATTTCACTTCTT   120
                                     ********* *************************

S.ph        CAAGTTTTTGTCCTACTATTATATCTTAATTTAAGTTGCTAGAGTACCTATTTTATTTTT   135
GI12.2      CAAGTTTTTGTCCTACTATTATATCTTAATTTAAGTTGCTAGAGTACCTATTTTATTTTT   137
GI12.9      CAAGTTTTTGTCCTACTATCATATCTTAATTTAAGTTGCTAGAGTACCTATTTTATTTTT   180
GI12.1      CAAGTTTTTGTCCTACTATTATATCTTAATTTAAGTTGCTAGAGTACCTATTTTATTTTT   137
GI12.10     CAAGTTTTTGTCCTACTATTATATCTTAATTTAAGTTGCTAGAGTACCTATTTTATTTTT   180
            ******************* ****************************************

S.ph        ATATTTATTACAAGGAAGAAACATAGTTCATCTTCTTCTATTCTTTTGTAGAAGATAAAA   195
GI12.2      ATATTTATTATAAGGAAGAAACATAGTTCATCTTCTTCTATTCTTTTGTAGAAGATAAAA   197
GI12.9      ATATTTATTATAAGGAAGAAACATAGTTCATCTTCTTCTATTCTTTTGTAGAAGATAAAA   240
GI12.1      ATATTTATTATAAGGAAGAAACATAGTTCATCTTCTTCTATTCTTTTGTAGAAGATAAAA   197
GI12.10     ATATTTATTATAAGGAAGAAACATAGTTCATCTTCTTCTATTCTTTTGTAGAAGATAAAA   240
            ********** *************************************************

S.ph        ATTAGAAAATAAAATCTTATATTCTTGGTTT-ATTTATTTTTATAAATTATTAAACAACA   254
GI12.2      ATTAGAAAATAAAATCTTATATTCTTGGTTT-ATTTATTTTTATAAATTATTAAACAACA   256
GI12.9      ATTAGAAAATAAAATCTTATATTCTTGGTTTTATTTATTTTTATAAATTATTAAACAACA   300
GI12.1      ATTAGAAAATAAAATCTTATATTCTTGGTTT-ATTTATTTTTATAAATTATTAAACA-AT   255
GI12.10     ATTAGAAAATAAAATCTTATATTCTTGGTTTTATTTATTTTTATAAATTATTAAACA-AT   299
            ******************************* *************************

S.ph        ATAATAACATATTTAATATAATGTCACAAGTGAGGTTTAAGAGGGTAGAGTGACAAGATC   314
GI12.2      ATAATAACATATTTAATATAATGTCACAAGTGAGGTTTAAGAGGGTAGAGTGACAAGATC   316
GI12.9      ATAATAACATATTTAATATAATGCCACAAGTGAGGTTTAAGAGGGTAGAGTGACAAGATC   360
GI12.1      ATAATAACATATTTAATATAATGCCACAAGTGAGGTTTAAGAGGGTAGAGTGACAAGATC   315
GI12.10     ATAATAACATATTTAATATAATGCCACAAGTGAGGTTTAAGAGGGTAGGGTGACAAGATC   359
            *********************** ************************ ***********

S.ph        TTACGTTTACCTTGTGAAGGTATAAAAGGTATTATTGAAAGACGTTCAACTCAAGTGCAA   374
GI12.2      TTACGTTTACCTTGTGAAAGTATAAAAGGTATTATTGAAAGACGTTCAACTCAAGTGCAA   376
GI12.9      TTACGTTTACCTTGTGAAGGTATAAAAGGTATTATAGAAAGACCTTCAACTCAAGTGCAA   420
GI12.1      TTACGTTTACCTTGTGAAGGTATAAAAGGTATTATTGAAAGACCTTCAACTCAAGTGCAA   375
GI12.10     TTACGTTTACCTTGTGAAGGTATAAAAGGTATTATTGAAAGACCTTCAACTCAAGTGCAA   419
            ****************** **************** ******* ****************

S.ph        CAAAACAAAGTATTCATGAAAAAGGAAATACAATAGTGAACAAAACATGTCAATTAATAA   434
GI12.2      CAAAACAAAGTATTCATGAAAAAGGAAATACAATAGTGAACAAAACATGTCAATTAATAA   436
GI12.9      CAAAACAAAGTATTTATGAAAAAGGAAATACAATAGTGAACAAAACATGTCAATTAATAA   480
GI12.1      CAAAACAAAATATTTATGAAAAAGGAAATACAATAGTGAACAAAACATGTCAATTAATAA   435
GI12.10     CAAAACAAAATATTTATGAAAAAGGAAATACAATAGTGAACAAAACATGTCAATTAATAA   479
            ********* **** *********************************************

S.ph        GAAAAGTAGTACAAACCATTCAGGAAAAAAGAGAGAATAATAACAATAATGAAATAATGC   494
GI12.2      GAAAAGTAGTACAAACCATTCAGGAAAAAAAAGAGAATAATAACAATAATGAAATAATGC   496
GI12.9      GAAAAGTAGTACGAACCATATAAGAAAAAAGAGAGAATAATAACAATAATGAAATAATGC   540
GI12.1      GAAAAGTAGTACAAACCATTCAAGAAA-AAAAAAGAATAATAACAATAATGAAATAATGC   494
GI12.10     GAAAAGTAGTACAAACCATTCAAGAGA-AAAAAAGAATAATAACAATAATGAAATAATGC   538
            ************ ******   * ** * ** * **************************

S.ph        GCTAACTGAAACACATTAGACAACAAATGGTATCAGATATCAAAAGCAAGAAATTACATA   554
GI12.2      GCTAACTGAAACACATTAGACAACAAATGGTATCAGATATCAAAAGCAAGAAATTACATA   556
GI12.9      GTTAACGGAAACACATTAGACAACAAATGGTATCAGATATCAAAAGCAAGAAATTACATA   600
```

```
GI12.1      GCTAACTGTAACACATTAGACAACAAATGGTATCAGATATCAAAAGCAAGAAATTACACA   554
GI12.10     GCTAACTGTAACACATTAGACAACAAATGGTATCAGATATCAAAAGCAAGAAATTACACA   598
            * **** * ************************************************* *

S.ph        TATAAGGCTAATATTACGAGAACACGGTGCTCCCAAACTCCCACTAAGTCTAATCCCGCT   614
GI12.2      TATAAGGCTAATATTACGAGAACATGGTGCTCCCAAACTCCCACTAAGTCTAATCCCGCT   616
GI12.9      TATAAGGCTAATATTACGAGAACACGGTGCTCCCAAACTCCCACTAAGTCTAATCCCGCT   660
GI12.1      TATAAGGCTAATATTACGAGAACACGGTGCTCCCAAACTCCCACTAAGTCTAATCCCGCT   614
GI12.10     TATAAGGCTAATATTACGAGAACACGGTGCTCCCAAACTCCCACTAAGTCTAATCCCGCT   658
            ************************ ***********************************

S.ph        GAAAAGTGAGTCAAAACTCAATTACCTACTAACATTCTACCTTATTCTGCAATCTCCACA   674
GI12.2      GAAAAGTGAGTCAAAACTCAATTACCTACTAACATTCTACCTTATTCTGCAATCTCCACA   676
GI12.9      GAAAAGTGAGTCAAAACTCAATTACCTACTAACATTCTACCTTATTCTGCAATCTCCACA   720
GI12.1      GAAAAGTGAGTCAAAACTCAATTACCTACTAACATTCTACCTTGTTCTGCAATCTCCACA   674
GI12.10     GAAAAGTGAGTCAAAACTCAATTACCTACTAACATTCTACCTTGTTCTGCAATCTCCACA   718
            ******************************************* ****************

S.ph        CCCTCCTATCTAATATCATGTCTTCAATCGGGAAGTTGAAGTTGTACCATGTCCTGTCTA   734
GI12.2      CCCTCCTATCTAATATCATGTCTTCAATCGGGAAGTTGAAGTTGTACCATGTCCTGTCTA   736
GI12.9      CCCTTCTATCTAACATCATGTCTTCAATCGGGAAGTTGAAGTTGTACCATGTCCTGTCTA   780
GI12.1      CTCTCCTATCTAATATCATGTCTTAAATCGGGAAGATGAAGTTGTACCATGTCCTGTCTA   734
GI12.10     CTCTCCTATCTAATATCATGTCTTAAATCGGGAAGATGAAGTTGTACCATGTCCTGTCTA   778
            * ** ******** ********** ********** ************************

S.ph        ATTACCTCTCTTCAATACTTCTTAAGTTTATCTTCACATTTTCGCATACCATCATATTCA   794
GI12.2      ATTACCTCTCTTCAATACTTCTTAAGTTTATCTTCACATTTTCGCATACCATCATATTCA   796
GI12.9      ATTACCTCTCTCCAATACTTCTTAAGTTTATCTTCACATTTTCGCATACCACCATATTCA   840
GI12.1      ATTACCTCTCTCCAATACTTCTTAAGTTTATCTTCACATTTTCGCATACCACCATATTCA   794
GI12.10     ATTACCTCTCTCCAATACTTCTTAAGTTTATCTTCACATTTTCGCATACCACCATATTCA   838
            *********** *************************************** ********

S.ph        ACCTCTCACACCTCCTCACTAGGCATCTT-----------------CATACGTCCAATTC   837
GI12.2      ACCTCTCACACCTCCTCACTAGGCATCTT-----------------CATACGTCCAATTC   839
GI12.9      ACCTCTCACACCTCCTCACTAGGCATCTTCATACACATTTCCTCTTCATACGTCCAATTC   900
GI12.1      ACCTCTCACACCTCCTCACTAGGCATCTTCATACACATTTCCTCTTCATACGTCCAATTC   854
GI12.10     ACCTCTCACACCTCCTCACTAGGCATCTTCATACACATTTCCTCTTCATACGTCCAATTC   898
            *****************************                 **************

S.ph        ATCTTAATCCCGCTTCTCTCATTTTATCCACCACAAAGACTATTCACATTTTATCCAAAA   897
GI12.2      ATCTTAATCCCGCTTCTCTCATTTTATCCACCACAAAGACTATTCACATTTTATCCGAAA   899
GI12.9      ATCTTAATCCCGCTTCTCTCATTTTATCCACCACAAAGACTATTCACATTTTATCCCAAA   960
GI12.1      ATCTTAATCCCGCTTCTCTCATTTTATCCACCACAAAGACCATTCACATTTTATCCCAAA   914
GI12.10     ATCTTAATCCCGCTTCTCTCATTTTATCCACCACAAAGACCATTCACATTTTATCCCAAA   958
            **************************************** *************** ***

S.ph        AATTCTTTTT-TTTAATCTT-ATCTCACTTAGTATGCAAACACATTAATAAGTTCTCAAA   955
GI12.2      AAATTCTTATTTTTAATCTTATCTCACTTTAGTATGCAGACACATTAATAAGTTCTCAAA   959
GI12.9      AATTCTTATTCT--TAATCTTATCTCACTTAGTATGCAAACACATTAATAAGTTCTCAAA   1018
GI12.1      AATTCTTATTCT--TAATCTTATCTCACTTAGTATGCAAACACATTAGTAAGTTCTCAAG   972
GI12.10     AATTCTTATTCT--TAATCTTATCTCACTTAGTATGCAAACACATTAGTAAGTTCTCAAG   1016
            ** *  *  * *   *   *        ********** ******** ***********

S.ph        CAATTGCAAAATCAATGTTATGAATACTACAATTATGGGTGTGTTAAGATTGTTTTTTTA   1015
GI12.2      CAATTGCAAAATCAATGTTATGAATACTACAATTATGGGTGTGTTAAGATTGTTTTTTTT   1019
GI12.9      CAATTGCAAAATCAATGTTATGAATACTATAATTATGGGTGTGTTAAGATTGTCTTTTTT   1078
GI12.1      CAATTGCAAAATCAATGTTATGAATACTACAGTTATGGGTGTGTTGAGATTGTTTTTTTA   1032
GI12.10     CAATTGCAAAATCAATGTTATGAATACTACAATTATGGGTGTGTTGAGATTGTTTTTTTA   1076
            ***************************** * ************* ******* *****

S.ph        AAAATAGCTTATAAGCTAAATGTCATAAAAGTCTATTTGGATTGACTTTTTATTTTTAAT   1075
GI12.2      AAAATAGCTTATAAGCTAAATGTCATAAAAGTCTATTTGGATTGACTTTTTATTTTTAAT   1079
GI12.9      TAAATAGCTTATAACCTAAATGT-ATAAAAGTCTATTTGGATTGACTTTTTATTTTTAAT   1137
GI12.1      AAAATAGCTTATAAGCTAGATGTCATAAAAGTCTATTTGGATTGACTTTTTATTTTTAAT   1092
GI12.10     AAAATAGCTTATAAGCTAGATGTCATAAAAGTCTATTTGGATTGACTTTTTATTTTTAAT   1136
             ************* *** **** ************************************
```

```
S.ph         TATTTTTTACTTATTTTTAACATTTTAGTTTAAAACTAAAGTGTTTAGCATTTTGACTTA   1135
GI12.2       TATTTTTTACTTATTTTTAACATTTTAGTTTAAAACTAAAGTGTTTAGCATTTTGACTTA   1139
GI12.9       TGTTTTTTACTTATTTTTAACATTTTAGTTTAAAACTAAAGTGTTTAGTATTTTGACTTA   1197
GI12.1       TATTTTTTACTTATTTTTAACATTTTAGTTTAAAACTAAAGTGTTTAGCATTTTGACTTA   1152
GI12.10      TATTTTTTACTTATTTTTAACATTTTAGTTTAAAACTAAAGTGTTTAGCATTTTGACTTA   1196
             * ********************************************** ***********

S.ph         TTTTTACCATTCCACTTTTCTTTTTCAGCTAACTAGCAATGTGAGACTTTATTCACATCT   1195
GI12.2       TTTTTACCATTCTACTTTTCTTTTTCAGCTAACTAGCAATGTGAGACTTTATTCACATCT   1199
GI12.9       TTTTTACCATTCTACTTTTCTTTTTCAGCCAACTAGCAATGTGAGACTTTATTCACATCT   1257
GI12.1       TTTTTACCATTCTACTTTTCTTTTTCAGCTAACTAGCAATGTGAGATTTTATTCACATCT   1212
GI12.10      TTTTTACCATTCTACTTTTCTTTTTCAGCTAACTAGCAATGCGAGATTTTATTCACATCT   1256
             ************ **************** *********** **** *************

S.ph         CCAACATCT--------------CTCTATAAATTTTCCATCAAGAAATACTAATTAGTTG   1241
GI12.2       CCAACATCT--------------CTCTATAAATTTTCCATCAAGAAATACTAATTAGTTG   1245
GI12.9       CCAACATCACAGTCTCTTTCTCTCTCTATAAAATTTCCATCAAGAAATACTAATTAGTTG   1317
GI12.1       CCAACTTCACAGTTTCTTTCTCTCTCTATAAAATTTCCATCAAGAAATACTAATTAGTTG   1272
GI12.10      CCAACTTCACAGTTTCTTTCTCTCTCTATAAAATTTCCATCAAGAAATACTAATTAGTTG   1316
             ***** **               ********* ***************************

S.ph         GATTAAATGAAGTAAGAAGTCGTTCGATTGGG      1273
GI12.2       GATTAAATGAAGTAAGAAGTCGTTCGATTGGG      1277
GI12.9       GATTAAATGAAGTAAGAAGTCGTTCGATTGGG      1349
GI12.1       GATTAAATGAAGTAAGAAGTCGTTCGATTGGG      1304
GI12.10      GATTAAATGAAGTAAGAAGTCGTTCGATTGGG      1348
             ********************************
```

***StGI.12* promoter proximal**

```
S.ph         AGTAAGAAGTCGTTCGATTGGGAACATGTTATTCCAGGATAAGTTATTACGAAAGGCCTA   60
GI12.4       AGTAAGAAGTCGTTCGATTGGGAACATGTTATTCCAGGATAAGTTATTACGAAAGGCCTA   60
GI12.12      AGTAAGAAGTCGTTCGATTGGGAACATGTTATTCCAGGATAAGTTATTACGAAAGGCCTA   60
GI12.2       AGTAAGAAGTCGTTCGATTGGGAACATGTTATTCCAGGATAAGTTATTACGAAAGGCCTA   60
GI12.10      AGTAAGAAGTCGTTCGATTGGGAACATGTTATTCCGGGATAAGTTACTACGAATGGCCTA   60
             *********************************** ********** ****** ******

S.ph         ACCCATCGGTGGCCCCCTAAAGTTGACACCATATTTTACTTAGACACTTCAATGGAGCGA   120
GI12.4       ACCCATCGGTGGCCCCCTAAAGTTGACACCATATTTTACTTAGACACTTCAATGGAGCGA   120
GI12.12      ATCCATCGGTGACCCCCTAAAGTTGACATCATATTTTACTTAGACATTTCAACGGAGCGA   120
GI12.2       ATCCATCGGTGACCCCCTAAAGTTGACATCATATTTTACTTAGACATTTCAACGGAGCGA   120
GI12.10      ACCCATTGGTGACCCTCTAAAGTTGACACCATATTTTACTTAGACACTTCAACGGAGCGA   120
             * **** **** *** ************ ***************** ***** *******

S.ph         TATTCATTTTAGACACCTCATGTAGGATTTCGCTGTGTCATTTTGATTATGTATATGAAA   180
GI12.4       TATTCATTTTAGACACCTCATGTAGGATTTCGCTGTGTCATTTTGATTATGTATATGAAA   180
GI12.12      TGTTCATTTTAGACACCTCATGTAGGGTCTCGTTGTGTCATTTTGATTATGTATATGAAA   180
GI12.2       TGTTCATTTTAGACACCTCATGTAGGGTCTCGTTGTGTCATTTTGATTATATATATGAAA   180
GI12.10      TGTTCATTTTAGACACCTTATGTAGGATCTCGTTGTGTCATTTCGATTATATATATGAAA   180
             * **************** ******* * *** ********** ****** *********

S.ph         TATTTTATTCCATCACTAATATATAAATGATGAGAAAAAATAGTTCCAGAACAACCTAAT   240
GI12.4       TATTTTATTCCATCACTAATATATAAATGATGAGAAAAAATAGTTCCAGAACAACCTAAT   240
GI12.12      TAATTTATCCCATTAA--ATATATAAATGATGAGATAAA-TAATCTCAGAACAACCTAAT   237
GI12.2       TAATTTATCCCATCACTAATATATAAATGATGAGATAAA-TAATCCCAAAACAACCTAAT   239
GI12.10      TAATTTATCCCATCACTAATATATAAATGATGAGATAAA-TAATCCCAAAACAACCTAAT   239
             ** ***** **** *   ***************** *** ** *  ** ***********

S.ph         ACCTCCGACTAAATACAAGATAAAATAGTCATACATTTTATCCCTAAGATTATTATGCCG   300
GI12.4       ACCTCCGACTAAATACAAGATAAAATAGTCATACATTTTATCCCTAAGATTATTATGCCG   300
GI12.12      ACCTCCAACTAAACACAAGATAAAATAGTCATTCATTTTATCTCTAAGATTATTATGCCA   297
GI12.2       ACCTCCGACTAAACACAAGATAAAATAGTTATATATTTTATCCCTAAAATTATTATGCCG   299
GI12.10      ACCTCCGACTAAACACAAGATAAAATAGTTATATATTTTATCCCTAAAATTATTATGCCG   299
             ****** ****** *************** **  ******** **** *********** 

S.ph         TATATCTCACACCGTAAATGAGAAAGTAGGTATGTATGTGATGATGAAGCAATTAGTCCA   360
GI12.4       TATATCTCACACCGTAAATGAGAAAGTAGGTATGTATGTGATGATGAAGCAATTAGTCCA   360
GI12.12      CATATCTCACACCATAAGTGAGAAAGTAGGTATGTATGTGATGATGAAGCAATTAGTCCA   357
GI12.2       TATATCTCACACCGTAAATGAGAAAGTAGGTATGTATGTGATGATGAAGCAATTAGTCCA   359
```

```
GI12.10     TATATCTCACACCGTAAATGAGAAAGTAGGTATGTATGTGATGATGAAGCAATTAGTCCA   359
             ************ *** ******************************************

S.ph        CTTGCTTGCCACATGGCCCTCTTAAGATCTGAGGAAAATATCATCGCCACGTATGCATTG   420
GI12.4      CTTGCTTGCCACATGGCCCTCTTAAGACCTGAGGAAAATATCATCGCCACGTATGCATTG   420
GI12.12     CTTGCTTGCCACATGGCCCTCTTAAGGTCTGAGGAAAATATCATCGCCACGTATGCATTA   417
GI12.2      CTTGCTTGCCACATGGCCCTCTTAAGATCTGAGGAAAATATCATCGCCACGTATGCATTA   419
GI12.10     CTTGCTTGCCACATGGCCCTCTTAAGATCTGAGGAAAATATCATCGCCACGTATGCATTA   419
            **************************  *******************************

S.ph        AGAAAACACAGCTAAAGATCTTCTTTCTGCTCCATATCTTTTTCTCTTCTTTAAACTCTT   480
GI12.4      AGAAAACACAGCTAAAGATCTTCTTTCTGCTCCATATCTTTTTCTCTTCTTTAAACTCTT   480
GI12.12     AGAAAACACAGTTAAAGATCTTCTTTCTGCTCCATATCTTTTTCTCTTCTTTAAACTCTT   477
GI12.2      AGAAAACACAGTTAAAGATCTTCTTTCTGCTCCATATCTTTTTCTCTTCTTTAAACTCTT   479
GI12.10     AGAAAACACAGTTAAAGATCTTCTTTCTGCTCCATATCTTTTTCTCTTCTTTAAACTCTT   479
            *********** ************************************************

S.ph        CTTCTCCTCCTTGTATTTTTTTCTCTCCAAATCCCTAAAATATCTTCCCTAAAATCCTTC   540
GI12.4      CTTCTCCTCCTTGTATTTTTTTCTCTCCAAATCCCTAAAATATCTTCCCTAAAATCCTTC   540
GI12.12     CTTCTCCTCCTTGTATTTTTTTCTCTCCAAATCCCTAAAATATCTTCCCTAAAATCTTTT   537
GI12.2      CTTCTCCTCCTTGTATTTTTTTCTCTCCAAATCCCTAAAATATCTTCCCTAGAATCTTTT   539
GI12.10     CTTCTCCTCCTTGTATTTTTTTCTCTCCAAATCCCTAAAATATCTTCCCTAAAATCTTTT   539
            *************************************************** **** **

S.ph        TT-TTTTTTCCAGATTTACCCTCACCAGTTTTCAGAATTCACATCTACGGTAGTTTACCA   599
GI12.4      TT-TTTTTTCCAGATTTACCCTCACCAGTTTTCAGAATTCACATCTACGGTAGTTTACCA   599
GI12.12     TTTTTTCTTCCAGATTTACCCTCACCAGTTTTCAGAATTCACATCTACGGTAGTTTACCA   597
GI12.2      TTTTTTCTTCCAGATTTACCCTCACCAGTTTTCAGAATTCACATCTACGGTAGTTTACCA   599
GI12.10     TTTTTTCTTCCAGATTTACCCTCACCAGTTTTCAGAATTCACATCTACGGTAGTTTACCA   599
            ** *** *****************************************************

S.ph        TTTTTTTTATTA--------AAATAAACTTTGTTCTAATAATTTTTTTCTGGGTTTCTTC   651
GI12.4      TTTTTTTTATTA--------AAACAAACTTTGTTCTAATAATTTTTTTCTGGGTTTCTTC   651
GI12.12     TTTTTTTTATTTTTTTTATTAAAATAAACTTTGTTCT-AATAATTTTTTCTGGGTTTCTTC   656
GI12.2      TTTTTTTTATTTTTTTTATTAAAATAAACTTTGCTCT-AATAATTTTTTCTGGGTTTCTTC   658
GI12.10     TTTTTTTTATTTTTTTTATTAAAATAAACTTTGCTCT-AATAATTTTTTCTGGGTTTCTTC   658
            ***********         *** ******** *** *  * ******************

S.ph        GTGCATAATATGTTATAGATGTTGAGCTGTATTTGTTTTTGAA--GGTTTTTTAAAAAAA   709
GI12.4      GTGCATAATATGTTATAGATGTTGAGCTGTATTTGTTTTTGAA--GGTTTTTTAAAAAAA   709
GI12.12     GTGCATAATATGTTGTAGGTGTTGAGCTGTTTTTTTGTTTTTGAAGGTTTTTTTTTAAAAA   716
GI12.2      GTGCATAATATGTTGTAGATGTTGAACTGTTTTTTTGTTTTTGAAGGTTTTTTTAAAAA-A   717
GI12.10     GTGCATAATATGTTGTAGATGTTGAACTGTTTTTTTGTTTTTGAAGGTTTTTTTAAAAA-A   717
            ************** *** ****** **** ***  ****  *  * *****   *** *

S.ph        GTTGTCGAACAGTTTTATTGTACTATTTTCGATTTTCTTTTTTTTAGGAATAAGAATTGAG   769
GI12.4      ATTGTCGAACAGTTTTATTGTACTATTTTCGATTTTCTTTTTTTTAGGAATAAGAATTGAG   769
GI12.12     ATTTTCGAACAGTTTTATTGTACTATTTTCGAT-T-TTTTTTTTAGGAATAAGAATTGAG   774
GI12.2      ATTGTCGAACAGTTTTATTGTACTATTTTCGAT-T-TTTTTTTTAGGAATAAGAATGGAG   775
GI12.10     ATTGTCGAACAGTTTTATTGTACTATTTTCGATTT-TT-TTTTTAGGAATAAGAATGGAG   775
             ** ***************************** *  * ***************** ***

S.ph        AAACAGTATATAATTGGAGAATTGATTATACTTCTAATTTATTTGAAAATATTTT-TTTT   828
GI12.4      AAACAGTATATAATTGGAGAATTGATTATACTTCTAATTTATTTGAAAATAATTTTTTTT   829
GI12.12     AAACAGTATATAATCGGAGAATTGATTATACTTCTAATTTACTTGAGAATAATTTA-TTT   833
GI12.2      AAACAGTATATAATCGGAGAATTGATTATACTTCTAATTTACTTGAGAATAATTTA-TTT   834
GI12.10     AAACAGTATATAATCGGAGAATTGATTATACTTCTAATTTACTTGAGAATAATTTA-TTT   834
            ************** ************************** **** **** ***  ***

S.ph        TTTTTTGACAATTTGTGCAAGTGATAATTTTCTGTTAATGGGAAAATGAAAAAAAAGAAG   888
GI12.4      TTTTTTGACAATTTGTGCAAGTGATAATTTTCTGTTAATGGGAAAATGAAAAAAAAGAAG   889
GI12.12     ATTTTTGACAATTTGTGCAAGTGATAGTTTTCTGTTAATGGGAAAATGAAAAAAA-AAAG   892
GI12.2      ATTTTTGACAATTTGTGCAAGTGATAGTTTTCTGTTAATGGGAAAATGAAAAAAAAAAAG   894
GI12.10     ATTTTTGACAATTTGTGCAAGTGATAGTTTTCTGTTAATGGGAAAATGAAAAAAA-AAAG   893
             ************************* ****************************  ***

S.ph        AAGTTTTTTGTT---------CCGTTTATTTTCAGATCATTTTGATGAATTAATACTTTT   939
GI12.4      AAGTTTTTTGTT---------CCGTTTATTTTCAGATCATTTTGATGAATTAATACTTTT   940
```

```
GI12.12      TTTTTTGTTCCCTTTATTTTTAGGAGAAAAAAGGGTTCATTTTGATGAATTAATACTTTT   952
GI12.2       TTTTTTGTTCCCTTTATTTTTAGGAGAAAAAAGGGTTCATTTTGATGAATTAATACTTTT   954
GI12.10      TTTTTTGTTCCCTTTATTTTTAGGAGAAAAAAGGGTTCATTTTGATGAATTAATACTTTT   953
                *** **              *   *      * ************************

S.ph         TTTTAAGCTTTCAAAAGGTATTAATTTAGTTGTGCATTTGTGTAACAGTTTGAAGTTTTT   999
GI12.4       TTTTAAGCTTTCAAAAGGTATTAATTTAGTTGTGCGTTTGTGCAACAGTTTGAAGTTTTT   1000
GI12.12      TTTTTAAGCGTCAAAAGGTATTAATTTAGTTGTGCATTTGTGTAACAGTTTGAAGATTTT   1012
GI12.2       TTTTAAGCTGTCAAAAGGTATTAATTTAGTTGTGCATTTGTGTAACAGTTTGAAGATTTT   1014
GI12.10      TTTTAAGCTGTCAAAAGGTATTAATTTAGTTGTGCATTTGTGTAACAGTTTGAAGATTTT   1013
             **** *    ************************* ****** ************ ****

S.ph         TTG--TTTTTTTTGTTGATGGTTTGTGCAAGTGAAATGAACTGCAAACGCTTTTTCCTGT   1057
GI12.4       TTG--TTTTTTTTGTTGATGGTTTGTGCAAGTGAAATGAACTGCAAACGCTTTTTCCTGT   1058
GI12.12      TTCT-TTTTTCTTGTTGATGATTTGTGCAAGTGAAATGAACTGCAAACACTTTTTCCTGT   1071
GI12.2       TTTTTTTTTTTTTGTTGATGATTTGTGCAAGTGAAATGAACTGCAAACACTTTTTCCTGT   1074
GI12.10      TTTT-TTTTTTTTGTTGATGATTTGTGCAAGTGAAATGAACTGCAAACACTTTTTCCTGT   1072
             **   ***** ********* *************************** ***********

S.ph         TAATGGGAAAAAATGGGGAAAAAAATTGTTCATTTTTTAGGAGAAAAAGGGTTCTTTTTA   1117
GI12.4       TAATGGGAAAAAATGGGGAAAAAAATTGTTCATTTTTTAGGAGAAAAAGGGTTCTTTTTA   1118
GI12.12      TAATGGGAAAAAATGGGA-AAAAAATTGTTCCTTTTTTAGGAGAAAAAGGGTCCTTTTTA   1130
GI12.2       TAATGGGAAAAAATGGGA-AAAAAATTGCTCCTTTTTTAGGAGAAAAAGGGTCCTTTTTA   1133
GI12.10      TAATGGGAAAAAATGGGA-AAAAAATTGTTCCTTTTTTAGGAGAAAAAGGGTCCTTTTTA   1131
             *****************  ********* ** ******************** *******

S.ph         GCTTTCAAAAGGTATTAATTTTGTTGTGCATTTGTTTAACTGTTTGATGATTTTTTATTT   1177
GI12.4       GCTTTCAAAAGGTATTAATTTTGTTGTGCATTTGTTTAACTGTTTGATGATTTTTTATTT   1178
GI12.12      GCTTTCAAAAGGTATTAATTTTGTTGTGCGTTTGTTTAACTGTTTGATGTTTTTTTATCT   1190
GI12.2       GCTTTCAAAAGGTATTAATTTTGTTGTGCATTTGTTTAACTGTTTGATGATTTTTTATTT   1193
GI12.10      GCTTTCAAAAGGTATTAATTTTGTTGTGCATTTGTTTAACTGTTTGATGATTTTTTATTT   1191
             ***************************** ******************* ******** *

S.ph         TTTGGTTTTGCAGGATAAAGTGGTGTATTATTATTGTCTTCTTCACTGTTTAATAGTGAA   1237
GI12.4       TTTGGTTTTGCAGGATAAAGTGGTGTATTATTATTGTCTTCTTCACTGTTAAATAGTGAA   1238
GI12.12      TTTGGTTTTGCAGGATAAAGTGGTGTATTATTATTGTCTTCTTCACTGTTAAATAGTGAA   1250
GI12.2       TTTGGTTTTGCAGGATAAAGTGGTGTATTATTATTGTCTTCTTCACTGTTAAATAGTGAA   1253
GI12.10      TTTGGTTTTGCAGGATAAAGTGGTGTATTATTATTGTCTTCTTCACTGTTAAATAGTGAA   1251
             ************************************************** *********

S.ph         ATCTACAACTGCCATAAGCTGGTTATAACATTAAAGGGAGTAGCTTGAAGCTCTAAGGTA   1297
GI12.4       ATCTACAACTGCCATAAGCTGGTTATAACATTAAAGGGAGTAGCTTGAAGCTCTAAGGTA   1298
GI12.12      ATCTACAACTGGCATAAGCTGGTTATAACATTAAAGGGACTAGCTTGAAGCTCTAAGGTA   1310
GI12.2       ATCTACAACTGGCATAAGCTGGTTATAACATTAAAGGGACTAGCTTGAAGCTCTAAGGTA   1313
GI12.10      ATCTACAACTGGCATAAGCTGGTTATAACATTAAAGGGACTAGCTTGAAGCTCTAAGGTA   1311
             *********** *************************** ********************

S.ph         ATTTTTCTTGATAAGATCCCTGGATCCCGTGGCA---------ATTTTCATTAAGGAGGT   1348
GI12.4       ATTTTTCTTGATAAGATCCCTGGATCCCGTGGCGGAGCCCAGAATTTTCATTAAGGAGGT   1358
GI12.12      ATTTTTCTTGATAAGATACCTGGACCCCGTGGCGGAGCCGG-AATTTTCATTAAGGAGTT   1369
GI12.2       ATTTTTCTTGATAAGATACCTGGACCCCGTGGCGGAGCCAG-AATTTTCATTAAGGAGTT   1372
GI12.10      ATTTTTCTTGATAAGATACCTGGACCCCGTGGCGGAGCCAG-AATTTTCATTAGGGAGTT   1370
             ***************** ****** ********          ********** **** *

S.ph         TCAAAATATGAAGTAAACATATGAAAAAGTTTAGGGGTTCAACACTTACCATATATACGT   1408
GI12.4       TCAAAATATGAAGTAAACATATGAAAAAGTTTAGGGGTTCAACACTTACCATATATACGT   1418
GI12.12      TCGAAATATGAAGTAAACATATGAAAAAGTCTAGGGGTTCAACATTTACCATATATACGT   1429
GI12.2       TCGAAATATGAAGTAAACATATGAAAAAGTCTAGGGGTTCAACATTTACCATATATACGT   1432
GI12.10      TCGAAATATGAAGTAAACATATGAAAAAGTCTAGGGGTTCAACATTTACCATATATACGT   1430
             ** *************************** ************* ***************

S.ph         AAAAAAAAAAAAAACCGTGTATAAACTGTATAATTATGAACCCCATTAACCTTACTAGTT   1468
GI12.4       AAAAAAA-AAAAAACCGTGTATAAACTGTATAATTATGAACCCCATTAACCTTACTAGTT   1477
GI12.12      AAA-----AAAAACCCGTGTATAAACTGTATAATTATGAACCCCATTAACCTTACTAGTT   1484
GI12.2       AAA-----AAAAACCCGTGTATAAACTGTATAATTATGAACCCCATTAACCTTACTAGTT   1487
GI12.10      AAA-----AAAAACCCGTGTATAAACTGTATAATTATGAACCCCATTAACCTTACTAGTT   1485
             ***     ***** **********************************************
```

```
S.ph        CCGCCCCTGCCTGGACCATCCTTGGATTGGGATCCAAATATGTTGAATTACAATATTTTG   1528
GI12.4      CCGCCCCTGCCTGGACCATCCTTGGATTGGGATCCAAATATGTTGAATTACAATATTTTG   1537
GI12.12     CCGCCCCTGCCTGGACCATCCTTGGATTGGGATCCAAATATGTTGAATTACAATATTTTG   1544
GI12.2      CCGCCCCTGCCTGGACCATCCTTGGATTGGGATCCAAATATGTTGAATTACAATATTTTG   1547
GI12.10     CCGCCCCTGCCTGGACCATCCTTGGATTGGGATCCAAATATGTTGAATTACAATATTTTG   1545
            ************************************************************

S.ph        ATCCTTGATTTGTAGCATTTTCTCGATCACTAAAATTCTTTGATTGTCAAACAGTTAT     1586
GI12.4      ATCCTTGATTTGTAGCATTTTCTCGATCACTAAAATTCTTTGATTGTCAAACAGTTAT     1595
GI12.12     ATCCTTGATTTGTAGCATTTTCTCGATCACTAAAGTTCTTTGGTTGTCAAACAGTTAT     1602
GI12.2      ATCCTTGATTTGTAGCATTTTCTCGATCACTAAAATTCTTTGGTTGTCAAACAGTTAT     1605
GI12.10     ATCCTTGATTTGTAGCATTTTCTCGATCACTAAAATTCTTTGGTTGTCAAACAGTTAT     1603
            ********************************** ******* ***************
```

**Fig. S2** Multiple sequence alignment of *StGI.12* promoter fragments cloned from *S. tuberosum* cv. 'Désirée' and retrieved for *S. tuberosum* Group Phureja (*S.ph*) from SpudDB. The *StGI.12* fragments extending from -2837 to -1586 bp and from -1586 to +59 bp were PCR amplified from the genomic DNA of 'Désirée' with the primer pair StGI1.2 -2837 FW and StGI.12 -1586 R, and StGI.12 -1586 FW and StGI.12 +59 R, respectively. Primer sequences are presented in Table S1. The fragments were cloned into pGEM-T-Easy and DNA of four recombinant clones was Sanger sequenced. The program Clustal Omega was used for multiple sequence alignment. 'Désirée'-specific CAREs predicted by PlantRegMap are in colour. The yellow-coloured region is a putative binding site of BBR-BPC family TF(s) PGSC0003DMG400003719 and/or PGSC0003DMG402029444, while the green-coloured region can be recognised by ERF-type TFs PGSC0003DMG400040046, PGSC0003DMG400002185, PGSC0003DMG400014541, and/or PGSC0003DMG400013402.
