# Supplementary material for: In Silico Characterization and Expression Analysis of GIGANTEA Genes in Potato
Source: Biochem Genet. 2022 Mar 11;60(6):2137–54. doi: 10.1007/s10528-022-10214-7 (PMC9617960; doi:10.1007/s10528-022-10214-7)
Supplement: Supplementary file 4 — Supplementary file4 (PDF 131 kb) [file 10528_2022_10214_MOESM4_ESM.pdf]

Biochemical Genetics

In Silico Characterization and Expression Analysis of *GIGANTEA* Genes in Potato

Flóra Karsai-Rektenwald, Khongorzul Odgerel, Jeny Jose[#], Zsófia Bánfalvi[*]

Genetic and Biotechnology Institute, Hungarian University of Agriculture and Life Sciences, 2100 Gödöllő, Szent-Györgyi A. u. 4, Hungary

[*] Corresponding author

E-mail: Banfalvi.Zsofia@uni-mate.hu

Table S5

CARE sequences in the potato *StGI.04* promoter

Biological process	ID	TF family	TF	Location (strand)	Binding site*
Circadian rhythm	PGSC0003DMG400021459<br>PGSC0003DMG400011048	MYB-related	REV1<br>REV8	-2051 (+)<br>-2051 (+)	AAAATATCT<br>AGAAAAATATCTCA<br>AGAAAAATATCT
	PGSC0003DMG401020784	bZIP	HY5	-2036 (-)	TGATGACGTGGC<br>TTTGCAGACGTGGCA
	PGSC0003DMG400027167	GATA	GATA1-like	-2484 (+)	ATGGTGATGATGATG<br>ATGGTGGGGATGGGG
Flower develop-ment	PGSC0003DMG400000008	M-type MADS	SOC1-like	-2906 (+)<br>-1985 (+)<br>-2456 (+)	TTTTTTTTTTTTTTTTTTT<br>GGTGTTTTTTTTTTTTTTTTTTTT<br>TCTCTTCTTCTCTCTCTTCA<br>TGTTTTTTTTTTTTTTTTTAT
	PGSC0003DMG400000027	MYB	MYB17	-2605 (+)	TTGGTAGGTGAG<br>GTGTTGGTGGGTGTG
	PGSC0003DMG400011048	MYB-related	REV8	-2051 (-)	AGATATTTT<br>AGATATTTTAGG
	PGSC0003DMG400024626	MIKC-MADS	CMB1-like	-795 (+)	CCATATATAG<br>GGTTCCTAATATAGATGC
Response to ABA	PGSC0003DMG400000088	bZIP	GBF3	-2036 (+)	TGCCACGTCAxC<br>TGCCACGTCTGCAAA
	PGSC0003DMG400002660<br>PGSC0003DMG400028121<br>PGSC0003DMG400008011<br>PGSC0003DMG400019455		ABI5<br>ABI5-like 2<br>ABI5-like 5	-2038 (-)<br>-2036 (-)<br>-2041 (+)	TGxTGACGTGGCA<br>ATTTGCAGACGTGGCATT<br>GGACACGTGGCA<br>TTTGCAGACGTGGCA<br>TGCCACGTGAxC<br>CTCAATGCCACGTCTGCA
			CPRF2	-2035 (-)	TGACGTGG<br>TGCAGACGTGGC
	PGSC0003DMG401020784		HY5	-2036 (-)	TGATGACGTGGC<br>TTTGCAGACGTGGCA
	PGSC0003DMG400019535	MYB	MYB96	-2610 (+)	TxGTTGGxxGTTG<br>ACAGTGTGTTGGTGGGTG
	PGSC0003DMG400000189<br>PGSC0003DMG400011048	MYB-related	REV6<br>REV8	-2050 (-)<br>-2051 (-)	AGATATTTT<br>AGATATTTTTC<br>AGATATTTTTCT
Tuber formation	PGSC0003DMG400004953	HB-other	POTH 20	-1988 (+)	CxCTCTxTCCCTGCCxCTxC<br>TCTTCTCTTC TCTCTCTCT

*The upmost sequence in each category is the consensus sequence. Coloured letters represent the highly conserved bases of the CAREs. The consensus sequences are according to PlantRegMap.
