# Supplementary material for: In Silico Characterization and Expression Analysis of GIGANTEA Genes in Potato
Source: Biochem Genet. 2022 Mar 11;60(6):2137–54. doi: 10.1007/s10528-022-10214-7 (PMC9617960; doi:10.1007/s10528-022-10214-7)
Supplement: Supplementary file 5 — Supplementary file5 (PDF 205 kb) [file 10528_2022_10214_MOESM5_ESM.pdf]

Biochemical Genetics

# *In Silico* Characterization and Expression Analysis of *GIGANTEA* Genes in Potato

**Flóra Karsai-Rektenwald, Khongorzul Odgerel, Jeny Jose[#], Zsófia Bánfalvi***

Genetic and Biotechnology Institute, Hungarian University of Agriculture and Life Sciences, 2100 Gödöllő, Szent-Györgyi A. u. 4, Hungary

*Corresponding author

E-mail: Banfalvi.Zsofia@uni-mate.hu

**Table S6**

**CARE sequences in the potato *StGI.12* promoter**

| Biological process | ID | TF family | TF | Binding site location (strand) | Binding site* |
|---|---|---|---|---|---|
| | | | | | AAAATATCT |
| **Circadian rhythm** | PGSC0003DMG400021459<br>PGSC0003DMG400011048 | MYB-related | REV1<br>REV8 | -1072 (+)<br>-1072 (+) | CCTAAAATATCTTC<br>CCTAAAATATCT |
| **Flower develop-ment** | | | | | CAACTTG |
| | PGSC0003DMG400008153 | bHLH | bHLH1 30 | -1230 (-) | GTCCACTTGCT |
| | | | | | TCAATTATTG |
| | PGSC0003DMG400002692 | HD-ZIP | ATHB 51 | -2975 (+) | TTGAATTATTGGC |
| | | | | | TTTTTTTTTTTTTTTTTT |
| | PGSC0003DMG400000008 | M-type MADS | SOC1 | -717 (-)<br>-1098 (+) | TCTTCTTTTTTTTTCATTTTCC<br>TCCTTGTATTTTTTTCTCTCC |
| | PGSC0003DMG401007392 | MIKC-MADS | FBP1 | -844 (-) | CCxAAAAAxGGAAA<br>CTAAAAAAAGAAAA |
| | | | | | AGATATTTT |
| | PGSC0003DMG400011048 | MYB-related | REV8 | -1072 (-) | AGATATTTTAGG |
| **Response to ABA** | | | | | TGCCACGTCAxC |
| | PGSC0003DMG400000088 | bZIP | GBF3 | -1181 (+)<br>-1219 (+) | CGCCACGTATGCATT<br>TGCCACATGGCCCTC |
| | | | | | TGxTGACGTGGCA |
| | PGSC0003DMG400002660 | | ABI5 | -1183 (-) | CAATGCATACGTGGCGAT |
| | PGSC0003DMG400008011 | | ABI5-like 5 | -1186 (+) | TGCCACGTGAxC<br>ATCATCGCCACGTATGCA |
| | PGSC0003DMG400028121 | | ABI5-like 2 | -1186 (-) | GGACACGTGGCA<br>ATCATCGCCACGTATGCA |
| | PGSC0003DMG401023951 | ERF | DREB 2A-like | -2045 (-) | GGxGGxGGxGxxGGCGGTG<br>TGAGGAGGTGTGAGAGGTTGA |
| | PGSC0003DMG400011048 | MYB-related | REV8 | -1072 (-) | AGATATTTT<br>AGATATTTTAGG |
| | | | | | CxCTCTxTCCCTGCCxCTxC |
| **Tuber formation** | PGSC0003DMG400004953 | HB-other | POTH 20 | -127 (+) | CTTACTAGTTCCGCCCCTGC |

*The upmost sequence in each category is the consensus sequence. Coloured letters represent the highly conserved bases of the CAREs. The consensus sequences are according to PlantRegMap
